# Redox-Switchable Single-Atom Catalyst Enables Efficient Aqueous Hydroxymethylfurfural Oxidation

**DOI:** 10.1021/acscatal.5c06280

**Published:** 2025-12-08

**Authors:** Jacky H. Advani, David Panáček, Petr Langer, Daniela Plachá, En Zhao, Shibo Xi, Zupeng Chen, Rajenahally V. Jagadeesh, Paolo Fornasiero, Giorgio Zoppellaro, Aristides Bakandritsos, Radek Zbořil

**Affiliations:** † Nanotechnology Centre, Centre for Energy and Environmental Technologies, 48207 VSB−Technical University of Ostrava, 17. listopadu 2172/15, Poruba, Ostrava 708 00, Czech Republic; ‡ Regional Centre of Advanced Technologies and Materials, Czech Advanced Technology and Research Institute (CATRIN), Palacký University Olomouc, Šlechtitelů 241/27, Olomouc 783 71, Czech Republic; § College of Chemical Engineering, 74584 Nanjing Forestry University, Longpan Road 159, Nanjing 210037, China; ∥ Institute of Sustainability for Chemicals, Energy and Environment (ISCE), 54759 Agency for Science, Technology and Research (A*STAR), 1 Pesek Road Jurong Island, Singapore 627833, Republic of Singapore; ⊥ Leibniz-Institut für Katalyse e.V., Albert-Einstein-Str. 29a, Rostock D-18059, Germany; # Department of Chemical and Pharmaceutical Sciences, Center for Energy, Environment and Transport Giacomo Ciamician, ICCOM-CNR Trieste Research Unit and INSTM Trieste Research Unit, 9315 University of Trieste, Trieste 34127, Italy

**Keywords:** iron single-atom catalyst, dimers, green oxidation, biomass valorization, 2,5-diformylfuran

## Abstract

The selective aerobic oxidation of biomass-derived 5-hydroxymethylfurfural (HMF) to 2,5-diformylfuran (DFF) is a pivotal step toward biobased polymers, pharmaceuticals, and fuels. Yet, most high-performance catalysts require noble metals and organic solvents and lose activity in water. Here, we report a robust and recyclable heterogeneous catalyst comprising mixed-valence single-atom iron dimers anchored on nitrogen-doped graphene acid (Fe–NGA), which mimics the powerful oxidation center in nonheme diiron oxidases. Spectroscopic and theoretical studies reveal a redox-flexible Fe^2+^/Fe^3+^ manifold that, under basic aqueous conditions, evolves into a Fe^3+^–Fe^4+^ ferryl species capable of highly selective proton-coupled two-electron oxidations. Fe–NGA achieves 97% HMF conversion with 95% DFF selectivity, a turnover frequency of 17.3 h^–1^, and a specific productivity of 12.5 mmol_DFF_ g_cat_^–1^ h^–1^ in pure water, surpassing state-of-the-art homogeneous and heterogeneous catalysts. The catalyst is stable with very low performance loss for at least six reactions. By merging such functionalities within a stable and reusable heterogeneous framework, Fe-NGA provides a benchmark earth-abundant catalyst for the effective oxidation of renewable feedstocks.

## Graphical abstract

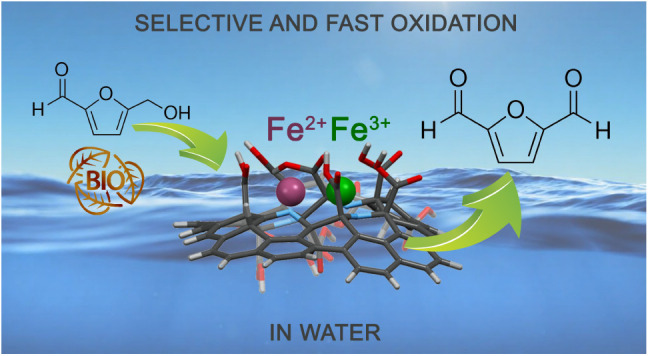

## 1 Introduction

The development of highly active and selective oxidation catalysts is essential for many important organic transformations, including green chemicals at the energy–environment interface.^1–5^ Among these, the oxidation of biomass-derived 5-hydroxymethylfurfural (HMF; one of the top 12 sustainable platform chemicals^6^) to 2,5-diformylfuran (DFF) is a flagship reaction in sustainable catalysis.^1,7^ DFF is a versatile C_6_ building block used in polymer manufacture, pharmaceuticals, antifungals, and fine chemicals, and is a key intermediate toward biobased aromatic replacements.^8–10^ Achieving this transformation effectively in water, without sacrificial oxidants or noble metals, is a central challenge for the integration of biomass upgrading into environmentally benign, scalable processes.^11^

Despite its apparent simplicity, the selective two-electron oxidation of HMF’s primary alcohol to DFF is challenging. The catalyst must oxidize the hydroxymethyl group without overoxidizing the aldehyde to the carboxylic acid or promoting side reactions at the α,β-unsaturated aldehyde moiety.^12–14^ In fact, the aldehyde group can get oxidized even in base alone, without a catalyst.^6,15^ Current state-of-the-art HMF-to-DFF catalysts still face major limitations related to sustainability (i.e., use of noble metals^13,16–18^ ), activity, selectivity, and recyclability. Homogeneous catalysts offer high activity, but are hard to recover and reuse, whereas heterogeneous analogues rarely reach comparable rates.^19,20^ For example, the benchmark oxovanadium complex reaches a mean turnover frequency (TOF) of 9.6 h^–1^ under full conversion conditions.^21^ Yet, heterogenizing such vanadium catalysts on carbon, reduces the activity to 5.7 h^–1^,^22^ retaining a specific productivity (SP) of ca. 6 mmol_DFF_ g_cat_^–1^ h^–1^.^16^ Efforts have also focused on photocatalysts, but, so far, have led to low activities (e.g., TOF < 1.5 h^–1^ or SP < 1.7 mmol_DFF_ g_cat_^–1^ h^–1^ for a single atom catalyst of Cu on carbon nitride,^23^ and a ZnIn_2_S_4_ plasmonic photocatalyst^12^).

Another bottleneck is the narrow solvent window, because most reported catalysts require predominantly aprotic organic media (Table S6). Bulk water generally promotes overoxidation to carboxylic acids and suppresses activity by displacing or blocking surface oxygen species with OH^–^/H_2_O.^1,18,24,25^ Interestingly, trace water has been reported to create a narrow optimum by tuning surface-bound H_2_O_2_ and reactive oxygen species (ROS), where for a CdZnS photocatalyst, adding ∼0.16 mL H_2_O to 10 mL acetonitrile raised DFF yield to 66%.^26^ However, yields declined sharply at higher water content, highlighting the persistent difficulty of maintaining both activity and selectivity in aqueous-rich media. Importantly, this challenge is not limited to photocatalysts that rely on free, nonadsorbed ROS, which are readily quenched in water. Even catalysts that activate O_2_ through lattice oxygen migration (Mars–van Krevelen pathways) or surface-bound oxygen intermediates are inhibited in high water concentrations because hydroxyl adsorption blocks the oxygen activation on the surface.^27^ Moreover, the mononuclear sites in SACs restrict access to cooperative and controlled oxygen activation pathways. In contrast, nature’s nonheme diiron oxidases provide an instructive blueprint: they exploit water and hydroxides as bridges in binuclear Fe centers, forming high-valent Fe^4+^=O (ferryl) intermediates that are both highly oxidizing and intrinsically selective in water.^28^ In these systems, water and hydroxyls are not poisons but essential participants. Translating such redox-cooperative motifs into robust, recyclable heterogeneous catalysts for operation in water represents an exciting but largely unexplored frontier in catalyst design.

Here we report an oxidation catalyst featuring preorganized single-atom iron dimers anchored on nitrogen-doped graphene acid (Fe–NGA), which stabilizes a redox-flexible Fe^2+^/Fe^3+^ manifold. Under the basic aqueous conditions, this catalyst forms an oxo-bridged Fe^3+^–Fe^4+^ ferryl species analogous to the reactive cores found in nonheme diiron enzymes.^28^ This rare active site structure enables selective, proton-coupled two-electron oxidation of HMF to DFF in pure water, achieving complete HMF conversion within 3 h, with a mean TOF of 17.3 h^–1^ and an SP of 12.5 mmol_DFF_ g_cat_^–1^ h^–1^, while maintaining DFF selectivity up to 95%. These performance metrics surpass even state-of-the-art homogeneous systems. Additionally, the catalyst demonstrates stability and recyclability for at least six reactions, with very small loss in activity. Fe–NGA thus achieves previously inaccessible high oxidizing power and aqueous-phase activity, offering a promising blueprint for sustainable oxidation processes and chemicals production in water.

## 2 Results and Discussion

### 2.1 Synthesis and Characterization of the Fe-NGA Catalyst

The NGA support was synthesized by first exfoliating commercially available fluorographite via sonication, followed by reaction with sodium azide.^29^ The nucleophilic azide ions attack the electrophilic centers associated with fluorine vacancies on fluorographene (FG).^30^ Subsequently, the azides disproportionate, leading to nitrogen atoms incorporated into the graphene plane, affording a 16 at.% *N*-doped graphene (NG, Figure 1a), as previously demonstrated.^31^ According to our earlier study, the resulting NG is a highly nitrogen-doped graphene containing numerous and large vacancies. HR-TEM and Raman spectroscopy revealed an intense D-band, indicative of sp^3^-type defect carbon sites that persist even after high-temperature treatment, confirming that these defects originate from the edges of vacancies and the periphery of the NG sheets.^31^ These structural features make NG particularly susceptible to oxidative treatment. Accordingly, the NG was oxidized using nitric acid to introduce a high density of carboxylic (COOH) groups, as validated by XPS results.^29^ The resulting NGA, rich in COOH functionalities, was then mixed with an aqueous solution of Fe­(NO_3_)_3_ to immobilize Fe cations toward the preparation of the Fe-NGA catalyst (Figure 1a).

**Figure 1:**
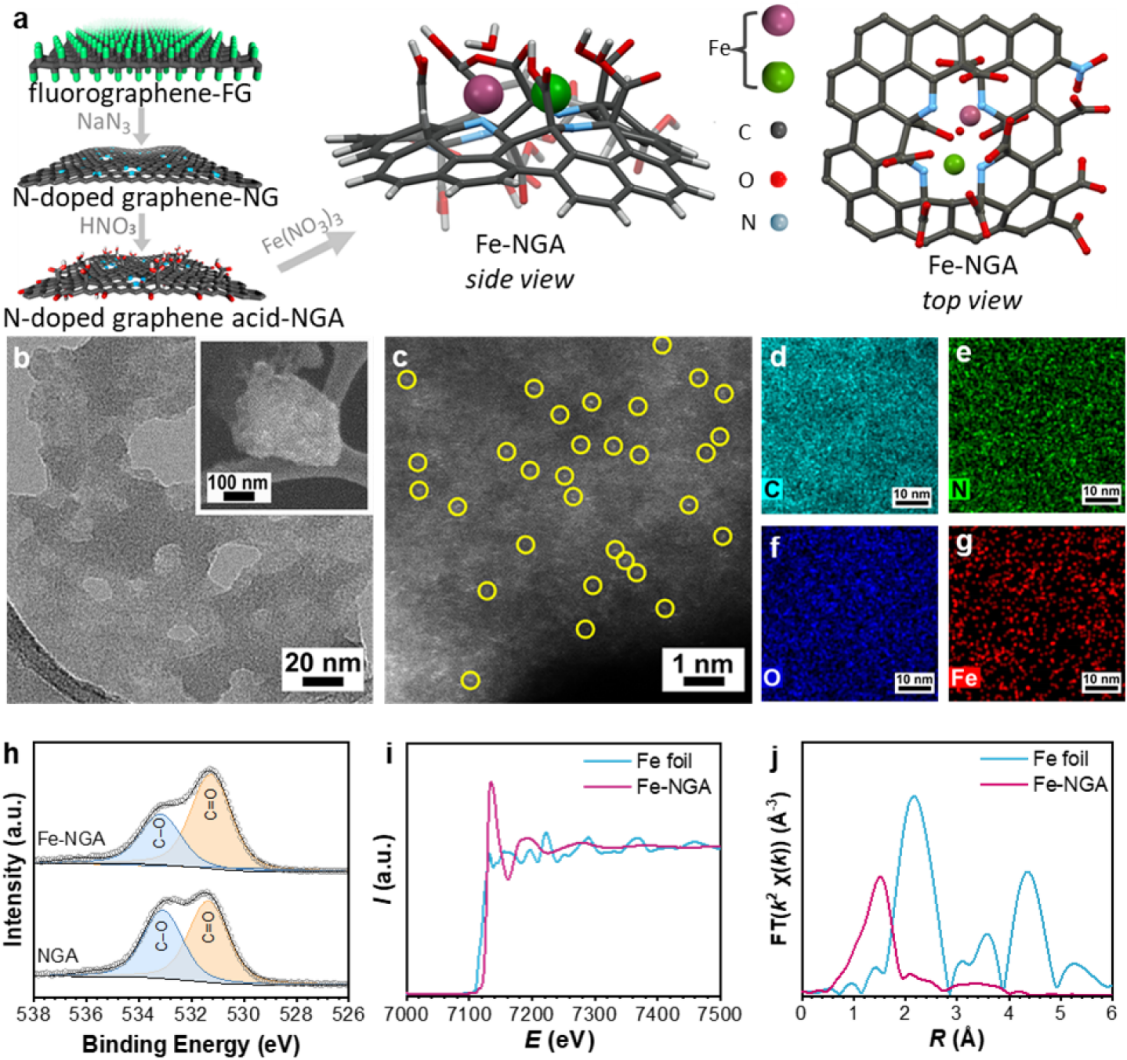
(a) Synthesis of Fe-NGA: iron atoms are coordinated with nitrogen and carboxyl/carboxylate groups. (b) HR-TEM and SEM (in the inset) micrographs of Fe-NGA, (c) HAADF-STEM image, and (d–g) EDS mapping of Fe-NGA. (h) HR-XPS spectra of the NGA support and Fe-NGA for the O 1s region. (i) XANES spectra and (j) R-space FT-EXAFS of Fe-NGA and Fe foil.

X-ray photoelectron spectroscopy (XPS) indicated extensive defluorination and nitrogen doping upon the transformation of FG to NG (as previously shown in detail^32^). Upon further transformation of NG to NGA, a substantial increase in oxygen-containing groups (as carboxyls) and a decrease in nitrogen content took place (as previously studied;^32^ XPS of the new batch of NGA is also provided here for direct comparison with the Fe-NGA, Figures S1 and S2). Fourier-transform infrared spectroscopy (FT-IR) of NGA showed the characteristic stretching band of the carboxylic groups at 1720 cm^–1^ (Figure S3). The spectrum also demonstrated two intense bands at ca. 1550 cm^–1^ and 1230 cm^–1^, both corresponding to skeletal vibrations of the *sp* ^2^ aromatic carbon rings of graphene.^33,34^ For the Fe-NGA, the intensity of the carboxylic groups decreased due to the interactions with the Fe cations, leading to partial ionization to carboxylates.^35^ As a result, the vibrations at ∼1600 cm^–1^ and ∼1420/1350 cm^–1^, corresponding to the asymmetric and symmetric stretching of the −CO_2_^–^ groups, respectively,^36^ increased markedly (Figure S3).

Transmission electron microscopy (TEM) revealed a flaky NGA/Fe-NGA morphology with lateral dimensions of ∼150 nm, a result of oxidative cleavage during nitric acid treatment (Figure 1b). High-angle annular dark-field scanning TEM (HAADF-STEM) showed no nanoparticles or clusters, only bright atomic-scale spots corresponding to individual Fe atoms (Figure 1c). Elemental mapping revealed the uniform dispersion of iron, with no observable local Fe aggregation (Figure 1d–g). The content of iron was 4 wt %, as determined by ICP analysis.

High-resolution XPS (HR-XPS) provided insight into chemical environments and bonding. In NGA and Fe-NGA, C 1s spectra contained sp^2^/sp^3^ carbon (284.7 eV), C–N (286.2 eV), and deprotonated/protonated carboxyls (287.2 and 288.6 eV; Figure S2a; Table S1). The O 1s spectra displayed two components associated with carboxylic groups: one corresponding to C=O (531.4 eV), and the other to C–OH (533.1 eV; Figure 1h; Table S3). The noticeable decrease in the C–OH area upon Fe ion incorporation confirms the interaction between the Fe ions and the carboxylic groups on the NGA support. This observation is in agreement with the deprotonation and higher charge density of carboxylate oxygens during carboxylic-carboxylate conversion and with the FT-IR results. For the Fe-NGA catalyst, XPS analysis showed 1.1 at.% in Fe (Figure S1b). The deconvolution of N 1s spectra for NGA and Fe-NGA showed three components, reflecting −N= (sp^2^), −NH– (sp^3^), graphitic, and oxidized nitrogen configurations (Figure S2b; Table S2). Fe 2p spectra revealed Fe^2+^ (710.4 eV) and Fe^3+^ (712.8 eV) species with characteristic satellites (Figure S2c), indicating partial Fe^3+^→Fe^2+^ reduction during immobilization. Such metal–graphene charge transfer and reduction is typically observed in such systems, as previously reported for Cu and Au cations using a nitrile functionalized graphene.^5,37^

The Fe K-edge XANES and EXAFS spectra clarify the oxidation state and local geometry of Fe in Fe-NGA. The absorption edge of Fe-NGA lies above that of Fe foil, confirming the oxidized state of Fe (Figure 1i). The pre-edge peak, arising from 1s → 3*d*/4p transitions, is weak in Fe(0) due to high symmetry, but pronounced in Fe-NGA, which indicates a low symmetry coordination environment, consistent with a distorted octahedral geometry (Figure 1i).^38,39^ Additionally, the postedge region exhibits dampened oscillations compared to Fe foil (Figure 1i), indicating that Fe is atomically dispersed rather than embedded in a crystalline, ordered local environment, as further confirmed by k-space EXAFS (Figure S4a).^40^ The FT-EXAFS is dominated by a first-shell Fe–N/O contribution at ∼2.02 Å with a coordination number of ∼6 (Figure 1j, Figure S4b, and Table S4). In the phase-uncorrected R-space plots there are no intense features beyond ∼2.5 Å, excluding long-range order and ruling out Fe nanoparticles or extended Fe–Fe networks (Figure 1j; see also k-space data in Figure S4a).^41^ A small second-shell contribution is captured by including a weak Fe···Fe scattering path at ∼2.86 Å in the fit with a coordination number of ∼1.2 (Table S4), implying spatially proximate single-atom Fe sites and not metallic Fe–Fe bonds or clustering. This improves the fit to a subtle shoulder near ∼2.4–2.5 Å in phase-uncorrected R.

A theoretical structural model (UHF/PM3tm) for the Fe-NGA system (Figure 1a and Figure S5) converged in excellent agreement with synchrotron and XPS results. In the model, the NGA plane contains a carbon divacancy, where two Fe cations are positioned in close proximity, with Fe–N (1.80–1.88 Å), Fe–O (1.93–2.16 Å), and Fe–Fe (2.51 Å) distances. The apical sixth coordination in the model is occupied by a water molecule. The double vacancy is selected as the most stable configuration in nitrogen-doped derivatives originating from fluorographene.^34,42^ The model is also in full agreement with the XPS analysis, containing 1 part −N= (sp^2^), 3 parts −NH– (sp^3^), very low graphitic nitrogen, and one-part oxidized nitrogen configurations (Figure S2b), as well as matching with the total carbon, oxygen, and nitrogen atoms. It is noted that some iron cations could also interact with carboxyl groups only, further above the plane of the NGA, as previously observed for other types of d-block metal cations.^29^

### 2.2 The Electronic Configuration of the Catalyst

To unveil the electronic spin configuration and gain further insights into the structure and properties of Fe-NGA, X-band EPR was performed. The EPR spectrum of neat NGA support in water (Figure 2a,h) showed one strong and isotropic resonance signal at *g*_eff_ = 1.998. The signal was unchanged, in both intensity and signal line shape, in a basic environment (0.1 M, K_2_CO_3_, pH = 11, Figure 2e,l). This EPR signal arises from the spin-containing defects located on a carbon center, which belongs to the NGA framework. This clearly indicates that the NGA support remained stable with respect to the spin defects concentration, under the reaction conditions used later for the catalytic reaction.

**Figure 2:**
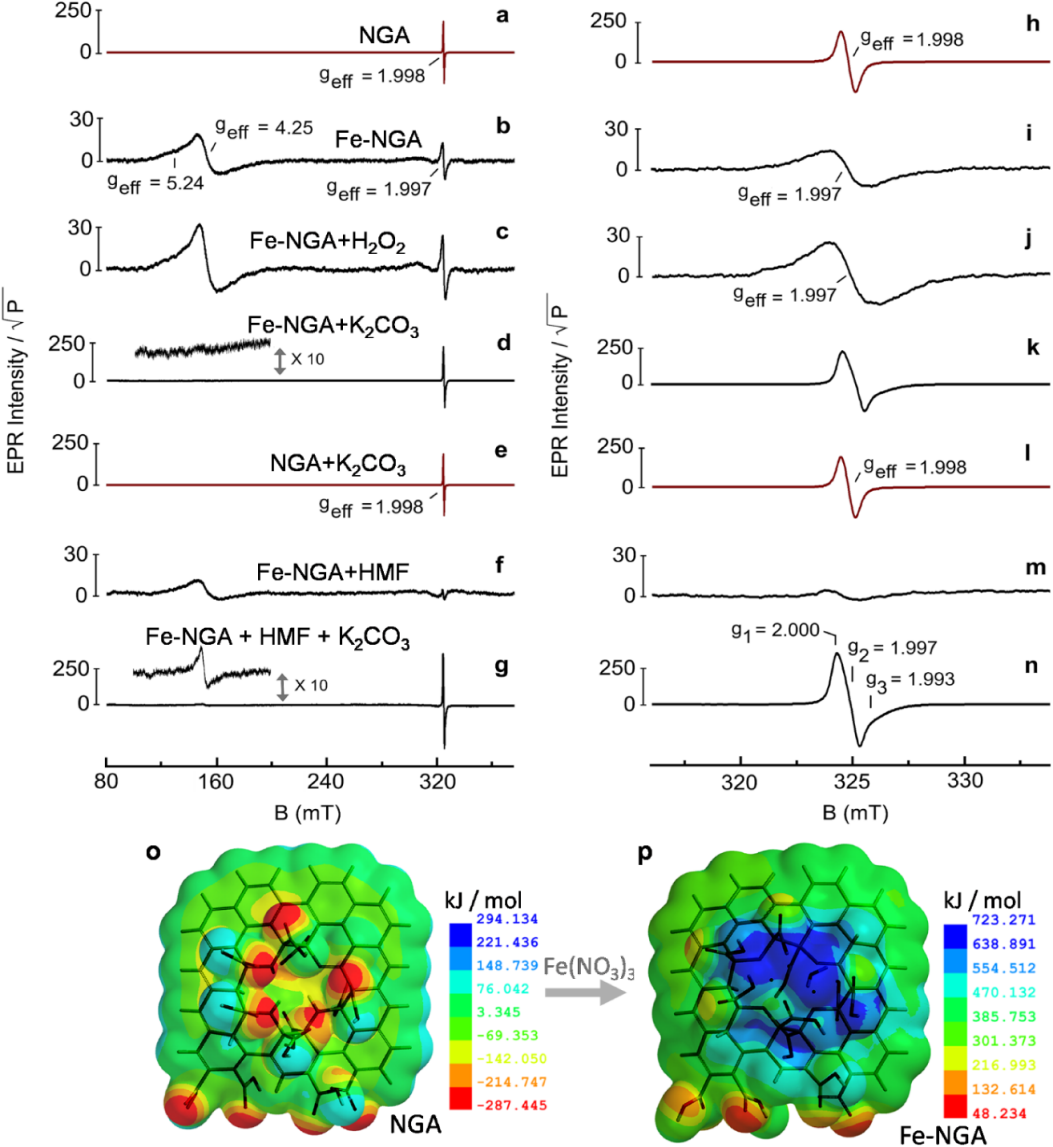
X-band EPR spectra recorded at *T* = 90 K and in ambient oxygen of (a, h) the neat NGA in water after 5 min sonication at 80 °C; (e, l) the neat NGA in the presence of K_2_CO_3_ (100 mM); (b, i) the Fe-NGA catalyst in neat water after sonication at 80 °C (5 min), and (c, j) after addition of H_2_O_2_ (30% w/w in water; 10 μL added to the 150 μL water suspension of NGA) and 5 min sonication at 80 °C; (d, k) the Fe-NGA catalyst in the presence of K_2_CO_3_ (100 mM); (f, m) the Fe-NGA catalyst in water and HMF (50 mM); (g, n) the Fe-NGA catalyst with K_2_CO_3_ (100 mM), water and HMF (50 mM). Experimental parameters for (a, h) 9.084 GHz and 0.6 mW; (b, i) 9.079 GHz and 0.9 mW; (c, j) 9.077 GHz and 0.9 mW; (d, k) 9.081 GHz and 0.2 mW; (e, l) 9.079 GHz and 0.9 mW; (f, m) 9.074 GHz and 0.6 mW; (g, n) 9.082 GHz and 0.9 mW. 0.6 mT modulation width, 30 ms time constant, 12 min acquisition time. The EPR intensities have been divided by the square root of the applied power. (o) Molecular electrostatic potential map surface of the NGA model (Table S7) obtained from RHF/PM3 calculation (C_63_H_33_N_5_O_24_, neutral, Heat of formation = −2349.23 kJ/mol) (p) Molecular electrostatic potential map of the Fe-NGA model (Table S8) obtained from UHF/PM3tm calculation (C_63_H_30_N_5_O_24_ × Fe^2+^Fe^3+^ × H_2_O, dication, Heat of formation = −4115.21 kJ/mol).

The EPR fingerprints of the Fe-NGA catalyst were substantially different from those witnessed in neat NGA. When Fe-NGA was suspended in neat water, the spectrum (Figure 2b) displayed two distinct resonance features. In place of the strong and narrow isotropic resonance signal at *g*_eff_ = 1.998 (ΔB_pp_ = 0.7 mT) in neat NGA, a much broader and weaker in intensity resonance feature (ΔB_pp_ = 1.7 mT) emerged at *g*_eff_ = 1.997 in Fe-NGA. The signal change of the spin-containing defects in the NGA framework is affected by the presence of the paramagnetic Fe cations, causing a signal broadening due to the dipolar magnetic interactions acting on spin centers of different natures (Fe, C-radicals). The strength of these interactions, and the broadening effects are inversely proportional to the distance (r) of the two different spin centers, ΔB_br_ ∝ *g* ^2^β^2^ **S**_Fe_.**S**_rad_/r^3^.^43^ The second resonance signal that appears in the low magnetic field region (*g*_eff_ > 4.00) corresponds to the middle-Kramer doublet (*m*_s_ ± 3/2) of high spin (*S* = 5/2) Fe^3+^ cations coordinated to the NGA backbone. The observed signal anisotropy indicates that the Fe^3+^ cations are entrapped in different coordination environments; a fraction of Fe^3+^ cations experience strong rhombic distortion, as given by the E/D ratio of the *zero-field-splitting* components (E/D ∼ 0.3, *g*_eff_ = 4.25), while the resonance shoulder around *g*_eff_ = 5.24 indicates the presence of bound Fe^3+^ cations with significantly weaker rhombic field (E/D ∼ 0.2). The notations D and E correspond to axial and rhombic *zero-field-splitting* terms of high-spin Fe^3+^, respectively. Moreover, the EPR spectra performed on the Fe-NGA sample in water with or without a sonication step at 80 °C revealed no net changes in the signal shape and intensities associated with the Fe^3+^ and radical centers, validating the stability of the Fe-NGA catalytic system, from the EPR signatures perspective, in the temperature range close to that used during HMF oxidation. Upon direct addition of H_2_O_2_ inside the EPR tube containing the water dispersion of Fe-NGA, the EPR spectrum showed an increase in the signal intensities of both the radical centers located on NGA, as well as the signal associated with the Fe^3+^ cations (Figure 2c,j). Comparison of the double integrated signal intensity of the overall Fe^3+^ resonances (B-field range of 100–200 mT) before and after the addition of hydrogen peroxide indicated that ∼30% of the Fe cations were present in NGA in the reduced form, as Fe^2+^. The higher Fe^3+^/Fe^2+^ ratio observed in EPR in comparison to that in XPS is ascribed to the different measurement conditions: EPR is performed in water, where oxidation of Fe^2+^ is commonly observed, whereas in XPS, the sample is measured in a dry state and under inert conditions. It should be noted that these Fe^2+^ centers can adopt either the high-spin configuration (*S* = 2, integer spin system), with large *zero-field-splitting* components (D, E > 0.3 cm^–1^), or can be present in the low-spin configuration (*S* = 0); both spin configurations, at X-band frequency, result into EPR silent species.

When Fe-NGA was dispersed in water with a base (10 μL of K_2_CO_3_ solution to reach a pH ∼ 11), and the solution heated at 80 °C for 5 min, followed by fast freeze-quench, a very strong, narrow (ΔB_pp_ = 1.0 mT) and anisotropic resonance signal is recorded (*T* = 90 K), expressing gtensor parameters at *g*_1_ = 2.000, *g*_2_ = 1.997, *g*_3_ = 1.993 (*g*_eff_ = 1.997) (Figure 2d,k). Moreover, no resonance signal attributable to Fe^3+^ cations was detected. The strong resonance fingerprint at *g*_eff_ = 1.997 differs substantially from the S = 1/2 weak signal associated with the carbon-based spin-containing defects seen in the neat NGA framework, both in the presence (Figure 2e,l) and absence (Figure 2a,h) of base. In addition, the appearance of such a signal (*g*_eff_ = 1.997, Figure 2d,k) was independent of the specific base used. Substituting K_2_CO_3_ with 0.05 M NaOH produced an identical resonance feature. This signal disappeared upon neutralizing the basic environment to pH 7, indicating that it is linked to the Fe spin-active species involved in a pH-dependent equilibrium. We suggest that such a strong *S* = 1/2 resonance signal, with small g-anisotropy, originates from the formation of a mixed-valence Fe^3+^-Fe^2+^ dimer structure, where the two iron centers are bridged by a μ-hydroxo group [Fe^3+^–OH-Fe^2+^]. In such a scenario, the *S* = 5/2 (Fe^3+^) and *S* = 2 (Fe^2+^) states interact antiferromagnetically, giving a radical-like *S* = 1/2 signature. Comparable signals in the *g* = 2.00 region, with radical-like feature and *S* = 1/2 spin configuration, have been observed in several dinuclear Fe complexes (μ-hydroxo) in which the mixed valence states (Fe^2+^/Fe^3+^) were conveniently produced by radiolytic reduction of the ferric dimers^44^ or by chemical conversion of μ-oxo into the μ-hydroxo systems, as those seen in diiron dipyrrin Pacman complexes.^45^ To study the catalytic potency of these Fe dimers in Fe-NGA to perform in oxidation reactions, we used HMF as a model substrate (Figure 3a) and monitored the changes in the EPR signals of Fe-NGA during the oxidation process.

**Figure 3:**
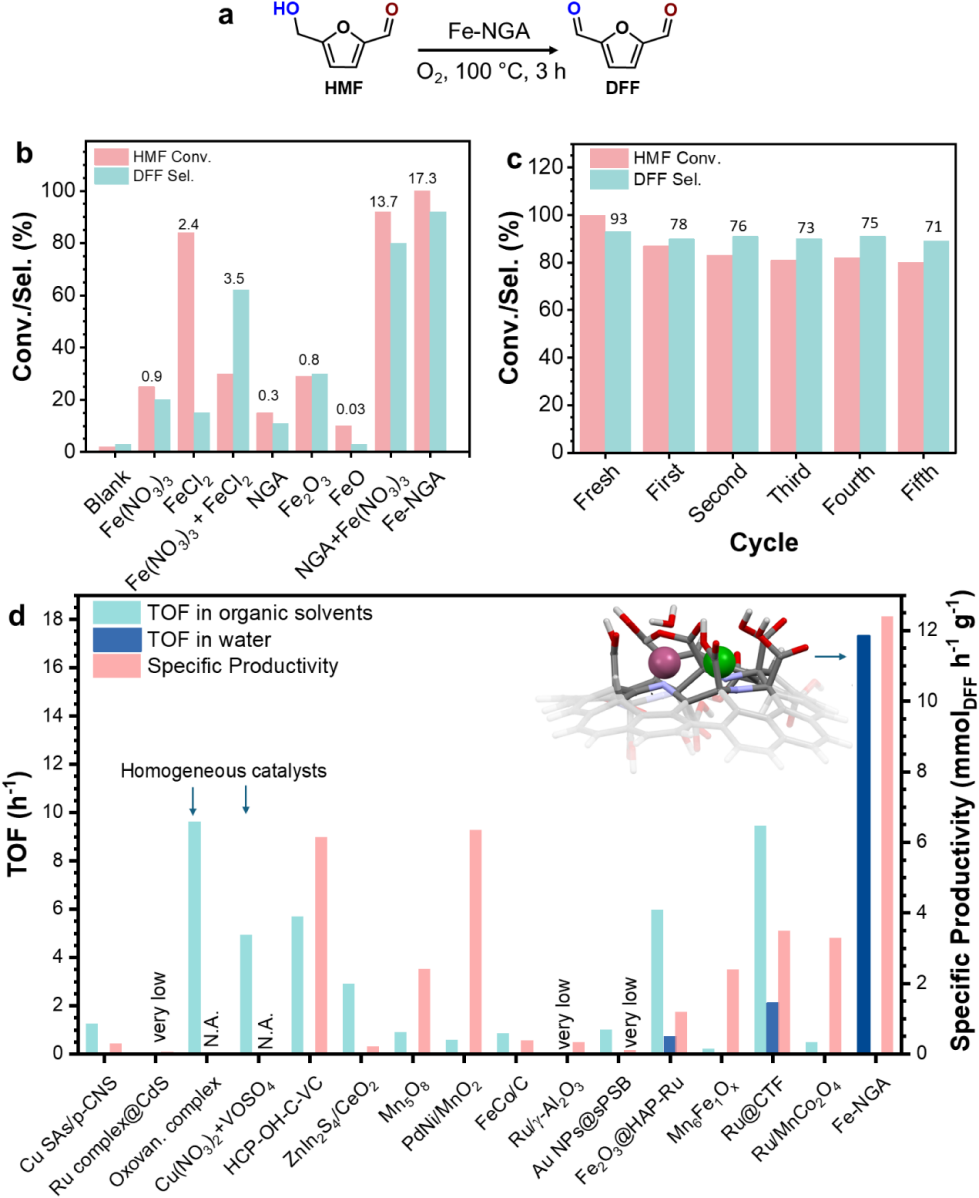
(a) HMF oxidation to DFF, (b) Catalytic performance benchmarking with control experiments, (c) Recyclability of the Fe–NGA catalyst in the oxidation of HMF to DFF. The GC yields of DFF for each cycle are indicated above the corresponding bars, and (d) comparison of the performance (TOF and SP) of previously reported catalysts with the Fe-NGA for HMF oxidation to DFF. Reaction conditions: 0.125 mmol HMF, 3.1 mg Fe-NGA (1.7 mol % Fe; HMF/metal molar ratio 56:1), 0.5 MPa O_2_, 0.25 mmol K_2_CO_3_, and 2 mL water. In the case of Fe-salts and other Fe-materials, the amount of catalyst corresponds to 1.7 mol % of Fe. Conversion and yields were determined by HPLC.

The EPR spectrum recorded after adding HMF to the aqueous Fe-NGA suspension and sonicated at 80 °C for 5 min (without base; Figure 2f,m) did not show the signal associated with the formation of the mixed-valent state Fe^3+^-μ–OH-Fe^2+^ system, confirming that the basicity is key for the Fe^3+^-μ–OH-Fe^2+^ generation. Furthermore, the resonance associated with the presence of high-spin Fe^3+^ centers became much weaker, as seen from the intensity of the signals evolving at *g*_eff_ = 4.25 and *g*_eff_ = 5.24. The weakened intensity, compared to the Fe^3+^ signal intensity seen in Figure 2b,i (without HMF, without base), indicates that HMF interacts with the Fe^3+^ metal centers, resulting in their reduction to Fe^2+^, which is EPR silent at X-band. However, only a small fraction of the total Fe in NGA appears to be active in the absence of base, as experimentally observed (*vide infra*). When the base is added, the electronic properties of the Fe-NGA system dramatically changed (Figure 2g,n), showing again a strong *S* = 1/2 resonance at *g*_*eff*_ = 1.997, signaling the Fe^3+^μOH-Fe^2+^ formation, complemented by a well-defined and sharp Fe^3+^ component (*g*_eff_ = 4.25). Taking all the information gained from EPR measurements and analysis, we suggest that in the Fe-NGA catalyst, the Fe-centers act cooperatively as Fe pairs during HMF oxidation.

The organization and electronic features of Fe-NGA mimic the behavior known in binuclear nonheme iron enzymes that activate oxygen in biocatalytic processes.^28^ In these natural systems, a strong radical signal that expresses small g-anisotropy is seen at *g* ∼ 2.00, displaying nearly identical fingerprints to those shown in Figure 2d,k,g,n. This signal is known to originate from a [Fe^3+^-μ-oxo/μ-hydroxo-Fe^4+^] or [Fe^3+^-bis μ-oxo-Fe^4+^] species formed upon O_2_ activation at the diferrous site. Such a signal, in the nonheme di-iron system ribonucleotide reductase R2 (RNR R2), has been termed intermediate X.^46–49^ A nearly identical radical signal, observed here for FeNGA in K_2_CO_3_ and K_2_CO_3_ + HMF around *g*_*eff*_ = 2.00, has also been reported for the tris­(3,5-dimethyl-4-methoxylpyridyl-2-methyl)­amine ligand chelating a di-iron center. In this case, a valence-localized [HO–Fe^3+^–O–Fe^4+^=O] open core is formed upon addition of OH^–^.^50^ Therefore, we suggest that Fe-NGA is capable of exhibiting high redox flexibility, forming the initial Fe dimer core (Fe^3+^-μ–OH-Fe^2+^ in neat base), and the Fe^3+^–(μ–OH)-O_2_–Fe^4+^ ferryl-containing species upon addition of the base and substrate in the presence of O_2_. The formation of the high-valent Fe­(IV) species during HMF catalysis should significantly enhance the oxidation process.

The formation in the Fe-NGA of a pocket prone to favor oxidation is also reflected in the computed molecular electrostatic potential maps from geometry-optimized models for neat NGA (RHF/PM3) and Fe-NGA (UHF/PM3tm) (Figure 2o,p). Significant changes in the electron density distribution upon Fe binding are observed. The initially negative (electron-rich, red color) areas around the C divacancy in the NGA plane shift to positive (electron-poor, blue color) regions, indicating that these sites are prone to interact with electron donors, for instance, with a substrate undergoing oxidation reaction.

### 2.3 Catalytic Performance

To exploit the unique oxidizing features of the Fe-NGA catalyst (cooperative oxygen activation in water and ferryl species formation), we evaluated the activity for the oxidation of HMF in more detail (Figure 3a). Reaction parameters (temperature, catalyst amount, base, and O_2_ pressure) were optimized (Table S5). The base (K_2_CO_3_) plays a critical role in the catalytic system. Its primary function is to facilitate the formation of the Fe^3+^-μ–OH-Fe^2+^ dimer species by providing hydroxide ions that bridge the Fe centers, as supported by spectroscopic and mechanistic analyses. To evaluate the effect of base, we also tested NaOH and K_3_PO_4_. NaOH promoted catalytic activity but also increased the formation of byproducts. K_3_PO_4_ resulted in lower HMF conversion. These observations indicated K_2_CO_3_ as the most appropriate base for the reaction. Under optimum conditions (100 °C, 0.25 mmol K_2_CO_3_, 0.5 MPa of O_2_), Fe-NGA showed a remarkable performance for the selective oxidation of HMF to DFF in pure water. The Fe-NGA catalyst with 4 wt % Fe loading achieved almost complete HMF conversion (97%) with 95% DFF selectivity, a TOF of 17.3 h^–1^, and a specific productivity of 12.5 mmol_DFF_ g^–1^ h^–1^ (Figure 3b,d). To probe the role of adjacent Fe centers for effective oxidation, a Fe-NGA catalyst with low Fe loading (2 wt %) was evaluated under identical conditions. In this case, the possibility of having adjacent Fe cations is significantly reduced due to the 2-fold lower Fe-loading. The total catalyst amount in the reaction was adjusted to maintain the same overall Fe content, as in the case of the 4 wt % Fe-NGA catalyst. Despite this adjustment, the catalytic activity significantly decreased, with a conversion of only 48% and a TOF of 8.3 h^–1^. This finding supports the EPR results, highlighting the crucial role of the Fe metal-ion synergy for achieving the highest performance.

To confirm that the activity and selectivity of the catalyst are not exclusively attributed to any of its individual components, control experiments were conducted (Figure 3b). A negligible catalytic activity was observed for NGA alone. Iron­(III) nitrate, in the absence of NGA, led to 23% HMF conversion, with 20% DFF selectivity and a TOF of 0.9 h^–1^. When ferrous chloride was used as the sole catalyst, the DFF selectivity was only 19%, giving a TOF of 2.4 h^–1^. Additionally, a mixture of iron­(II) and iron­(III) salts was tested, which resulted in 30% HMF conversion, 62% DFF selectivity, and TOF of 3.5 h^–1^. These outcomes show that freely diffusing Fe ions do not reproduce the activity-selectivity balance of Fe-NGA and underscore the need for preorganized Fe sites on NGA to enable controlled O_2_ activation. When the selectivity toward DFF was lower than in the optimized conditions, product analysis revealed the presence of intermediate oxidation products, such as HMFCA and trace amounts of FFCA. Several additional unidentified peaks also appeared in the chromatograms, especially under harsher reaction conditions. These signals likely correspond to products formed via well-known secondary pathways, including degradation, condensation of HMF or its intermediates, and polymerization (humins). Their formation is consistent with the complex reaction network typically associated with HMF oxidation. Another control experiment highlighted the importance of the immobilization of Fe^3+^ cations on the NGA support and of the stable binding of Fe under turnover. This experiment involved the addition of the Fe­(NO_3_)_3_ salt together with NGA. Unlike the case of adding only Fe­(NO_3_)_3_, the presence of NGA restored performance to levels comparable with Fe-NGA, highlighting the role of support in anchoring Fe under turnover and creating the active environment. The catalyst exhibited good recyclability; however, a decrease in HMF conversion was observed mainly in the first cycles (1st cycle 12.5%; second cycle 5%, third cycle 2%, fifth cycle 1%, Figure 3c). The activity loss is ascribed to small catalyst losses during the recovery steps, due to its high hydrophilicity and dispersibility in water (the solvent of the reaction). XPS analysis of the spent catalyst (Figure S8, ESI) revealed no significant structural changes or loss of active metal sites. Both the atomic percentages and the high-resolution Fe 2p spectra remained unchanged compared to the fresh catalyst.

### 2.4 Evaluation of Results in the Context of the State-of-the-Art

To correlate the performance of the Fe-NGA catalyst in context to the state-of-the-art, we compared the TOF and SP values (Figure 3d and Table S6). Homogeneous catalysts, such as oxovanadium complexes or VOSO_4_/Cu­(NO_3_)_2_ demonstrated some of the highest TOF for HMF oxidation to DFF.^21,51^ The oxovanadium complex achieved a TOF of 9.6 h^–1^ (Table S6, entry 3),^21^ while VOSO_4_/Cu­(NO_3_)_2_ displayed a TOF of 4.9 h^–1^ (Table S6, entry 14).^51^ However, the inherent challenges of catalyst separation and nonrecyclability associated with homogeneous systems have led to the development of heterogeneous catalysts. In this regard, vanadium complexes heterogenized on carbon supports showed promising results, achieving a production rate of 6.1 mmol_DFF_ g^–1^ h^–1^; however, with a quite low TOF of 5.7 h^–1^ (Table S6, entry 4).^22^ Several Ru-based heterogeneous systems have also been explored for this transformation.^17,18,25,52^ For example, Ru supported on γ-Al_2_O_3_ exhibited poor DFF selectivity (21.2%), despite a decent HMF conversion of 91.2%, likely due to overoxidation and other side reactions (Table S6, entry 10).^17^ On the other hand, Ru supported on covalent triazine frameworks (Ru@CTF) delivered a remarkable performance with a TOF of 9.4 h^–1^ and an SP of 3.5 mmol_DFF_ g^–1^ h^–1^, highlighting the advantage of tailored porous supports in achieving improved selectivity (Table S6, entry 12).^18^ Fe_2_O_3_@HAP-Ru reached full conversion with a TOF of 5.9 h^–1^ and an SP of 1.2 mmol_DFF_ g^–1^ h^–1^, underscoring the synergistic effect between iron oxide and ruthenium on hydroxyapatite (Table S6, entry 13).^25^ Ru/MnCo_2_O_4_ also delivered 98.3% conversion and complete selectivity for DFF, although with a low TOF of 0.5 h^–1^ (Table S6, entry 16).^52^ Gold-based catalysts, such as Au NPs@sPSB, showed moderate activity with a TOF of 1.0 h^–1^ and SP of 0.1 mmol_DFF_ g^–1^ h^–1^. However, the lower DFF selectivity (80%), possibly due to competing oxidation pathways, remains a drawback (Table S6, entry 11).^24^ PdNi supported on MnO_2_ achieved full conversion and excellent selectivity (99%) within 1 h, with a SP of 6.3 mmol_DFF_ g^–1^ h^–1^, albeit a modest TOF of 0.8 h^–1^ (Table S6, entry 8).^16^ Among non-noble catalysts, Mn_5_O_8_ and Mn_6_Fe_1_Ox achieved TOF values of 0.9 h^–1^ and ∼0.2 h^–1^, respectively, suggesting that while selectivity remains high (>94%), the activity is substantially lower than homogeneous catalysts (Table S6, entries 7 and 15).^53,54^ FeCo/C attained full conversion with excellent selectivity (99%), but its TOF and SP remained comparatively low (0.8 h^–1^ and 0.4 mmol_DFF_ g^–1^ h^–1^, respectively) (Table S6, entry 9),^55^ further illustrating the limitations in the activity of earth-abundant transition metal-based catalysts.

While heterogeneous and homogeneous catalysts have shown varying degrees of success, photocatalysts have gained significant attention for their potential in renewable energy and environmental applications. However, with respect to HMF-to-DFF oxidation, their performance remains suboptimal. For instance, S-scheme heterojunctions between ZnIn_2_S_4_ with sulfur vacancies and CeO_2_ have been explored for HMF oxidation to DFF, achieving a production rate (SP) of 0.9 mmol_DFF_ g^–1^ h^–1^ (Table S6, entry 6).^56^ Similarly, oxygen-doped ZnIn_2_S_4_ nanosheets with atomic-scale edge steps and lattice defects showed improved performance, achieving an SP of 1.6 mmol_DFF_ g^–1^ h^–1^ (Table S6, entry 5).^12^ A Ru complex supported on CdS quantum dots catalyzed HMF photo-oxidation to DFF, showing very low activity with a TOF of 0.03 h^–1^ and an SP of 0.05 mmol_DFF_ g^–1^ h^–1^ (Table S6, entry 2).^13^ Additionally, a photocatalyst comprising Cu–N_4_ and C–S–C as dual active sites supported on carbon nitride produced DFF with an SP of 0.3 mmol_DFF_ g^–1^ h^–1^ and a TOF of 1.2 h^–1^ (Table S6, entry 1).^23^

Despite these advances, three constraints recur across the literature: (i) overoxidation (suppressing DFF selectivity), (ii) low turnover frequencies under practical conditions, and (iii) the reliance on organic solvents. Particularly critical is the third constraint, as crude HMF streams contain water from fructose dehydration, and, in most cases, even trace amounts of H_2_O often degrade DFF selectivity and/or catalyst activity^18,24,25,52^ (see also Figure 3d for comparative activities in organic solvents and in water). In contrast, the Fe-NGA catalyst demonstrates unprecedented performance in pure water with 97–100% HMF conversion, 93–95% selectivity to DFF, a TOF of 17.3–17.4 h^–1^, and an SP of ∼12.5 mmol_DFF_ g^–1^ h^–1^. These figures not only surpass most noble-metal and homogeneous benchmarks in activity per active metal site but also demonstrate robust compatibility in aqueous-phase, effectively addressing a key bottleneck in sustainable HMF upgrading. We attribute this performance to the NGA framework’s ability to stabilize redox-flexible Fe–Fe dimers, which form the highly active catalytic center (ferryl-based iron dimer) in a basic aqueous environment. This, in turn, activates O_2_ cooperatively with high selectivity and rates, even in water. Moreover, to assess the scalability of the Fe-NGA system, the oxidation of HMF was conducted at a 10-fold higher substrate concentration under unoptimized conditions. Remarkably, complete HMF conversion and 96% DFF selectivity were achieved after 12 h, demonstrating that the catalyst retains excellent activity and selectivity even at higher substrate loadings. This result further highlights the robustness and potential industrial applicability of Fe–NGA for aqueous-phase aerobic oxidation. Collectively, these results position Fe–NGA as a practical blueprint for high-rate, selective aerobic oxidations directly in water.

### 2.5 Reaction Mechanism

Considering the obtained results, the presence of adjacent Fe cations (Fe^2+^ and Fe^3+^) in a bridged configuration [Fe^3+^-μ–OH-Fe^2+^], and the emergence of activated oxygen species under turnover [Fe^3+^-(O_2_)­(μ–OH)-Fe^4+^], the possible mechanism was explored. The two Fe sites are labeled as Site (1) and Site (2), as shown in Figure 4 (with additional information on intermediates in Figure S9). In the NGA framework, two closely interacting Fe centers can be present as a statistical combination of Fe^3+^-Fe^2+^, Fe^3+^-Fe^3+^, and Fe^2+^-Fe^2+^ active pairs in the resting state. Note that the Fe^3+^-Fe^3+^ and Fe^2+^-Fe^2+^ combinations, in the presence of base, cannot produce the strong radical-like signal observed in Figure 2d,k, because they are known to give EPR silent spectra.^46^ Therefore, two pathways are possible. The one shown in Figure 4a involves the formation of the spin-active dimers [Fe^3+^-μ–OH-Fe^2+^] and [Fe^3+^-(O_2_)­(μ–OH)-Fe^4+^]. The other pathway, starting from Fe^3+^-Fe^3+^ and Fe^2+^-Fe^2+^ couples expressing activate intermediates [Fe^3+^-(μ–OH)_2_-Fe^3+^] and [Fe^4+^-(O_2_)-Fe^4+^], is shown in Figure S9. In the aerobic oxidation of HMF to DFF, oxygen species such as superoxide radical (O_2_^•–^), peroxides (O_2_^–^), singlet oxygen (^1^O_2_), or hydroxyl radical (^•^OH) are thought to play key roles in the oxidation process. Generally, O_2_ acts as the terminal oxidant; the alcohol is initially dehydrogenated on the metal catalyst, which undergoes reduction (−CH_2_OH of HMF being oxidized to −CHO in DFF), and then O_2_ reoxidizes the catalyst. However, in Fe-NGA the activated oxygen specie (peroxide) bound to the Fe-centers directly acts in the HMF oxidation process. From the Fe^3+^-Fe^2+^ pair in the resting state, the addition of base rapidly forms intermediate I (Fe^3+^-(μ–OH)-Fe^2+^, *S* = 1/2) (Figure 4a), which activates an oxygen molecule and transforms into the intermediate II (Fe^3+^-(μ–OH)-(O_2_)-Fe^4+^), the hydroxo-peroxo intermediate (*S* = 1/2). These intermediates (I) and (II) give identical EPR signals as experimentally observed in Figure 2d,k. The HMF substrate then interacts with the active Fe^3+^-Fe^4+^ site (see also Figure S10, Monte Carlo simulations), delivering two electrons and two protons to the Fe bound peroxo-specie, triggering the release of water molecule and formation of a ferryl intermediate (Fe^4+^=O, intermediate III). This provides the first evidence under turnover of an uncoupled Fe–Fe system containing Fe^3+^–OH and Fe^4+^=O centers. The ferric center (Fe^3+^–OH) gives sharp and isotropic EPR signatures seen in Figure 2g, with *g* = 4.25, while the Fe^4+^=O site is EPR silent. A second HMF molecule then reacts with the high-valent Fe^4+^=O, in another proton-coupled two-electron transfer, releasing a second water molecule, generating intermediate (IV), Fe^3+^–OH, and Fe^2+^. These sites reform the bridged Fe^3+^/Fe^2+^ dimeric unit through the excess in solution of OH^–^anions, restarting the catalytic cycle back to intermediate I. Note that the reaction step I → II, which involves the oxygen binding and activation, is endothermic (PM6 method), and requires supply of thermal energy (*T*) (Figure 4b). This is consistent with our experimental evidence that the oxidation reaction to proceed needs both high temperature (100 °C) and O_2_ pressure, so to express effective substrate oxidation. Moreover, in the Fe^3+^-Fe^2+^ mixed valent spin configuration, the Fe^3+^ site is expected to provide the center interacting with the −CHO moiety of HMF (Figure S10), directing the HMF alcoholic residue (−CH_2_–OH) toward the Fe^2+^ site, involved in O_2_ binding and activation. Thus, the dimeric Fe-centers act synergistically to enhance the catalytic activity and selectivity of the Fe-NGA.

**Figure 4:**
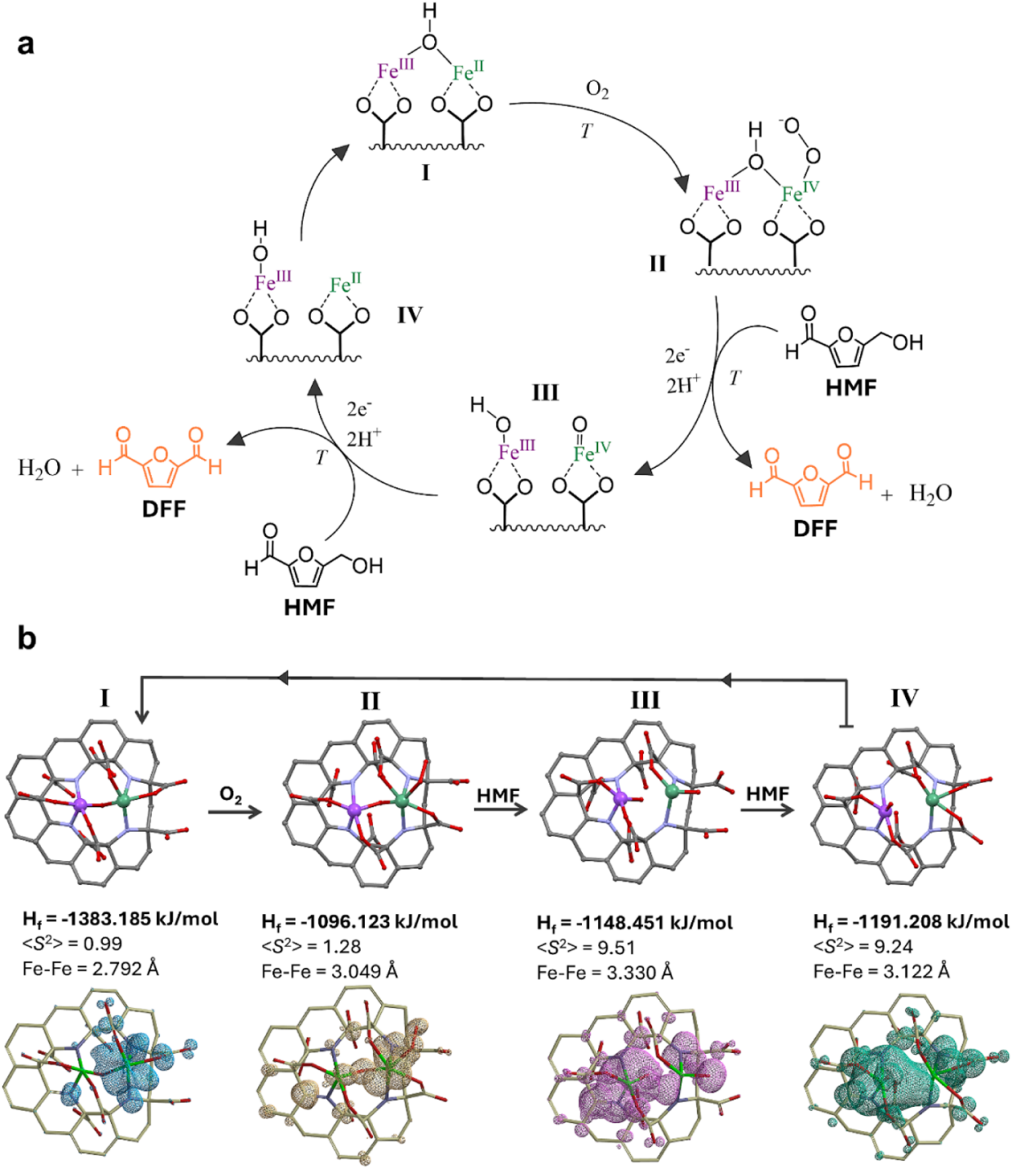
(a) Proposed reaction mechanism (main pathway) for the oxidation of HMF by Fe-NGA catalyst in a basic environment. (b) The computational models (UHF/PM6) show the Fe-NGA intermediates involved in the HMF catalysis, highlighting the variation of Fe–Fe distances (Å) within the catalytic cycle. H_f_ indicates Heat of Formation energy (kJ/mol) and < *S* ^2^> the expectation value. Spin density isosurfaces (0.002 IsoVal) are shown in the bottom structures.

The mechanism is compared to nonheme diiron enzymes to illustrate the formation of high-spin species; however, there is currently no direct evidence that HMF is cooperatively activated by both iron centers in the Fe^3+^-μ–OH-Fe^2+^ dimer. The proposed mechanism shows sequential oxidation steps mediated by the dimer, with both iron atoms necessary for catalytic activity, but it does not imply simultaneous cooperative activation of the substrate. Determining whether cooperative activation occurs will require further mechanistic investigation.

## 3 Conclusion

We have developed a robust oxidation catalyst comprising redox-flexible single-atom iron dimers stabilized on nitrogen-doped graphene acid. The Fe-NGA catalyst integrates enzyme-like active center features with the stability of heterogeneous catalysts, enabling selective aerobic oxidation of an important biomass-derived platform chemical under mild conditions in pure water. Comprehensive spectroscopic analyses revealed that under catalytic turnover, Fe-NGA mimics nonheme diiron oxidases by generating a high-valent oxo-bridged Fe^3+^–Fe^4+^ intermediate. This enables efficient, proton-coupled two-electron oxidations with high rates and remarkable selectivity, even in pure water, surpassing state-of-the-art systems. Thus, Fe-NGA catalyzed the complete HMF transformation to DFF with 93% selectivity, and a turnover frequency of 17.4 h^–1^, when the reaction was performed at 1 MPa of O_2_ (entry 4, Table S5). Mechanistic studies reveal that the cooperative action of adjacent Fe centers is essential for O_2_ activation and substrate oxidation, with the NGA support providing the precise coordination environment required to stabilize redox-switchable Fe^2+^/Fe^3+^/Fe^4+^ states. Importantly, this study introduces a previously unreported μ-hydroxo-bridged Fe^2+^–Fe^3+^ active-site architecture on a graphene-based support, formed in the presence of base, which serves as the true catalytically competent structure. This structurally defined and mechanistically validated diiron motif represents a new class of heterogeneous active centers that emulate enzymatic redox cooperation while operating in fully aqueous conditions. Such a discovery bridges biological and synthetic oxidation chemistry, offering a conceptual and structural advance in the design of earth-abundant, sustainable oxidation catalysts. This cooperative, redox-flexible manifold opens previously inaccessible mechanistic pathways in heterogeneous oxidation catalysis, offering a remarkable paradigm in aerobic oxidations with heterogeneous synthetic catalysts. This work offers a blueprint for developing next-generation heterogeneous systems for the green valorization of renewable feedstocks.

## 4 Experimental Details

### 4.1 Synthesis of Fe-NGA Catalyst

NGA was synthesized as previously described.^29^ 0.5 g of fluorographite was dispersed in 30 mL of DMF, followed by 24 h of sonication to ensure uniform dispersion. Then, 3 g of NaN_3_ was introduced, and the mixture was transferred to a round-bottom flask with a condenser and stirred at 130 °C for 3 days. The resulting product was subjected to purification by sequential washes with DMF, acetone, ethanol, water, and hot water, with each wash followed by centrifugation at 14000 rcf. A portion of this *N*-doped graphene was then oxidized by treatment with 45% HNO_3_ at 100 °C for 24 h in a glass flask with a condenser. The oxidized material was further purified by repeated washes with hot water and then dialyzed using a cellulose membrane (14 kDa cutoff). Finally, the oxidized nitrogen-doped graphene acid was mixed with an aqueous Fe­(NO_3_)_3_ solution and stirred at room temperature for 24 h. The resulting material was then washed with water and freeze-dried to obtain the Fe-NGA catalyst.

### 4.2 Catalytic Tests

The catalytic oxidation of the HMF was carried out in a 25 mL pressure reactor. The reactor was charged with 0.125 mmol HMF, 3.1 mg Fe-NGA catalyst (1.7 mol % Fe, HMF/metal molar ratio was 56:1), 0.25 mmol K_2_CO_3_, and 2 mL water, and the reaction mixture was sonicated for 5 min. The reaction was performed under a constant O_2_ pressure (typically 0.5 or 1 MPa) and maintained at the desired reaction temperature (100 °C) for a specific time (typically 3 h) with a stirring rate of 700 rpm. The reaction was quickly terminated by cooling the reactor to room temperature in an ice bath, and aliquots were taken from the mixture for product analysis by HPLC, as described in the Supporting Information. Larger scale testing was performed by increasing all reagents by 10-fold, except of the amount of water due to reactor limitations.

Additional experimental details are available in the Supporting Information file (Chemicals, Characterization, Product analysis from the catalytic reaction, Supplementary results figures and tables).

## Supplementary Material



## Data Availability

The data that support the findings of this work are openly available in Zenodo under the same title.

## Notes

Materials and methods; Figure S1: the XPS survey spectra of NGA support and Fe-NGA catalyst; Figure S2: the HR-XPS of the NGA support and Fe-NGA catalyst for the spectral regions of C 1s, N 1s, and Fe 2p; Figure S3: FT-IR spectra of NGA and Fe-NGA; Figure S4 reports the XAS analysis; Figure S5: the geometry optimized structures of NGA support and the Fe-NGA catalyst; Figure S6: calibration curves for HMF and for DFF; Figure S7: a representative chromatograph of product analysis for the HMF oxidation; Figure S8: the XPS analysis of the reused Fe-NGA catalyst; Figure S9: the other possible reaction mechanism for HMF oxidation by the Fe-NGA catalyst; Figure S10: the interaction of HMF with Fe-NGA in the presence of intermediate [II]; Table S1: comparative deconvoluted areas for C 1s region; Table S2: comparative deconvoluted areas for N 1s region; Table S3: comparative deconvoluted areas for O 1s region; Table S4: the structural parameters for Fe-NGA catalyst; Table S5: reaction optimization studies; Table S6: the comparative overview of the Fe-NGA with previously reported catalysts for HMF oxidation; Table S7: the Cartesian coordinates for the NGA system; Tables S8–S12: the Cartesian coordinates for the Fe-NGA system in different models (PDF)
