# Supplementary material for: Redox-Switchable Single-Atom Catalyst Enables Efficient Aqueous Hydroxymethylfurfural Oxidation
Source: ACS Catal. 2025 Dec 8;15(24):21057–68. doi: 10.1021/acscatal.5c06280 (PMC12723681; doi:10.1021/acscatal.5c06280)
Supplement: Supplementary file 1 [file cs5c06280_si_001.pdf]

Supporting Information

Redox-Switchable Single-Atom Catalyst Enables Efficient Aqueous Hydroxymethylfurfural Oxidation

Jacky H. Advani,[a] David Panáček,[a,b] Petr Langer,[a] Daniela Plachá,[a] En Zhao,[c] Shibo Xi,[d] Zupeng Chen,[c] Rajenahally V. Jagadeesh,[a,e] Paolo Fornasiero,[f] Giorgio Zoppellaro,[a,b*] Aristides Bakandritsos,[a,b*] Radek Zbořil[a,b*]

[a]Nanotechnology Centre, Centre for Energy and Environmental Technologies, VSB–Technical University of Ostrava, 17. listopadu 2172/15, Poruba, Ostrava 708 00, Czech Republic

[b]Regional Centre of Advanced Technologies and Materials, Czech Advanced Technology and Research Institute (CATRIN), Palacký University Olomouc, Šlechtitelů 241/27, Olomouc 783 71, Czech Republic

[c]College of Chemical Engineering, Nanjing Forestry University, Longpan Road 159, Nanjing 210037, China

[d]Institute of Sustainability for Chemicals, Energy and Environment (ISCE, Agency for Science, Technology and Research (A*STAR); 1 Pesek Road Jurong Island, Singapore, 627833, Republic of Singapore

[e]Leibniz-Institut für Katalyse e.V., Albert-Einstein-Str. 29a, D-18059 Rostock, Germany.

[f] Department of Chemical and Pharmaceutical Sciences, Center for Energy, Environment and Transport Giacomo Ciamician, ICCOM-CNR Trieste Research Unit and INSTM Trieste Research Unit, University of Trieste, Trieste, Italy

E-mails: giorgio.zoppellaro@upol.cz; a.bakandritsos@upol.cz; radek.zboril@upol.cz

1. Materials and methods

1.1. Chemicals

All chemicals were of analytical grade and used as received. 5-Hydroxymethylfurfural (HMF, 99%, Sigma-Aldrich), 5-hydroxymethyl-2-furancarboxylic acid (HMFCA, >99%, Sigma-Aldrich), 2,5-diformylfuran (DFF, >99%, Sigma-Aldrich), 5-formyl-2-furoic acid (FFCA, >99%, Sigma-Aldrich), 2,5-furandicarboxylic acid (FDCA, 99%, Sigma-Aldrich), graphite fluorinated polymer (>61 wt.% F-GF, Sigma-Aldrich), sodium azide (NaN_3, 99%, Millipore Sigma), dimethylformamide pure (DMF, Lachner), absolute ethanol (Penta), nitric acid (HNO_3, 65%, Lachner), acetone (99%, Millipore Sigma), $Fe(NO_3)_3·9H_2O$ (p.a., Merck), Acetonitrile (CHROMASOLV® gradient grade, for HPLC, ≥99.9%, Sigma-Aldrich) and Ammonium formate (99%, Penta) were purchased from different companies. Ultrapure water (18.2 MΩ) was used for the preparation of all the solutions and for the EPR measurements.

1.2. Characterization

The fine morphological characteristics of the catalyst were determined with high-resolution transmission electron microscopy (TEM) at 80 kV accelerating voltage on FEI Titan G2 60-300 transmission electron microscope equipped with an X-FEG electron gun, objective-lens image spherical aberration corrector, and a ChemiSTEM EDS detector. The structure of the catalyst was also analyzed with a TEM JEOL 2010 with LaB_6 type emission gun, operating at 160 kV with a resolution of 0.19 nm. For the sample preparation, a very dilute dispersion of catalyst (~0.1 mg mL^{-1}) was prepared by sonication and deposited on a carbon-coated copper grid and analyzed after drying for 24 h at room temperature. STEM-elemental mapping, used to determine the EDS pattern and the distribution of elements, was performed using HAADF-STEM (high-angle annular dark-field scanning transmission electron microscopy) on a FEI Titan HRTEM microscope operating at 80 kV.

Continuous wave electron paramagnetic resonance (EPR) spectra were recorded on a JEOL JES-X-320 spectrometer (JEOL, Tokyo, Japan) operating at X-band frequency (~9.08 GHz) and equipped with a variable-temperature controller (He, N_2) ES-CT470 apparatus. Highly pure quartz tubes (Suprasil, Wilmad, ≤0.5 OD) were used as sample holders, and accuracy in the experimentally determined g-values was obtained by using a Mn(II)/MgO standard (JEOL standard, g = 2.00105). The cavity quality factor (Q) was kept above 6000 in all measurements. The filling factors were kept constant at 150 μL. Experimental temperature was set to T = 100 K in all measurements, with a modulation frequency of 100 kHz, a time constant of 30 ms, and an acquisition time of 8 minutes. The Fe-NGA concentration during measurements was kept in the range of 5.3-5.8 mg/mL in H_2O. To probe the interaction between the Fe-NGA catalyst and K_2CO_3 base (K_2CO_3, 0.1 M in water), hydrogen peroxide (30% w/w), and the substrate (HMF), different solutions were prepared. To 200 μL of the Fe-NGA catalyst dispersed in ultrapure water, either (a) 10 μL of hydrogen peroxide (30%) stock solution, or (b) 10 μL of K_2CO_3 stock solution together with 10 μL of HMF in water (50 mM) was added. The mixtures were sonicated in a pre-heated ultrasound water bath for at least 5 min. Afterwards, 150 μL of the mixture was quickly transferred to quartz EPR tubes, freeze-quenched in a dry-ice acetone bath, and the EPR spectra were recorded at 100 K.

The metal content of fresh and reused catalyst was determined with ICP-MS (Agilent 7700x, Agilent, Japan). A weighted amount of sample from the catalyst (on a 0.01 mg read-out balance, Kern ABT 220-5DNM) was digested with nitric acid in a microwave digester followed by dilution with water. The mixture was centrifuged to precipitate solid residues, and the upper half of the supernatant was used for Fe determination.

X-ray photoelectron spectroscopy (XPS) analyses were performed using a Thermo Scientific K-Alpha instrument equipped with an Al Kα source (10 mA, 14 kV) and operating at 1486.8 eV during the measurement. The pass energy was set to 30 eV for high-resolution analysis. Charge shift was corrected by adjusting the binding energy C 1s to 284.7 eV. The data processing was performed using the

commercial Avantage software (Thermo Fisher Scientific Inc.). For the fitting procedure, a Shirley background has been used, and Lorentzian–Gaussian ratio was fixed at 30%.

X-ray absorption fine structure (XAFS) spectra and X-ray absorption near edge structure (XANES) spectra of Fe K-edge were performed at the X-ray absorption fine structure for catalysis beamline (covers Fe K-edge 7.11 keV) of the Singapore Synchrotron Light Source (SSLS), Singapore. The EXAFS oscillations $\chi(k)$ were extracted and analyzed by the Demeter software package.

Theoretical calculations of the geometry-optimized NGA and Fe-NGA models were performed by semiempirical (RHF/PM3 and UHF/PM3tm) methods. The computational software Spartan 10 (v. 1.1.0), Wavefunction Inc., Irvine CA. 92612, USA, was used for the theoretical treatments.

1.3. Product analysis from the catalytic reaction

The sample after the catalytic reaction was diluted with ultrapure water and filtered through a PTFE filter (0.22 μm). The reaction products were analyzed by HPLC (Waters Alliance, separation module e2695) equipped with a photodiode array detector (Waters PDA detector 2998) using an XBridge C18 column (3.5 μm 4.6 mm×100 mm). The mobile phases were acetonitrile (eluent A) and 0.5 mM ammonium formate in ultrapure water (eluent B) with a flow rate of 0.8 mL min^{-1}. The following gradient elution was used: 0-0.2 min 90% B, 0.5 – 4.0 min 40% B and 4.5 – 13.0 min 90% B. The injection volume was 5 μL. The column temperature was set at 40 °C. The maximum absorption was found for HMF (λ=284 nm), FDCA (λ=263 nm), FFCA (λ=286 nm), HMFCA (λ=250 nm) and DFF (λ=289 nm). HMF conversion, DFF selectivity, TOF and specific productivity are defined as follows:

$$C_{HMF}\ (\%) = \frac{n_{HMF\,initial} - n_{HMF\,final}}{n_{HMF\,initial}} \times 100\% \quad (1)$$

$$S_{DFF}\ (\%) = \frac{n_{DFF}}{n_{HMF\,initial}} \times 100\% \quad (2)$$

$$TOF\ (h^{-1}) = \frac{n_{DFF}}{n_{metal} \times t\ (h)} \quad (3)$$

$$Specific\ Productivity\ (SP) = \frac{n_{DFF}}{g_{catalyst} \times t\,(h)} \quad (4),$$

where n represents the mmoles of the HMF, the DFF or the mmoles of Fe in the Fe-NGA catalyst and $g_{catalyst}$ represents the mass of the whole catalyst added in the reaction.

Regarding the recycling procedure, after completion of each reaction cycle, the reaction mixture was extracted with ethyl acetate three times to remove DFF and any organic residues. The aqueous phase containing the hydrophilic catalyst was then reused directly for the next cycle by adding a fresh amount of HMF substrate under the same optimized reaction conditions. This procedure was repeated for five consecutive cycles.

Supplementary results

2. Catalyst characterization

2.1. XPS analysis

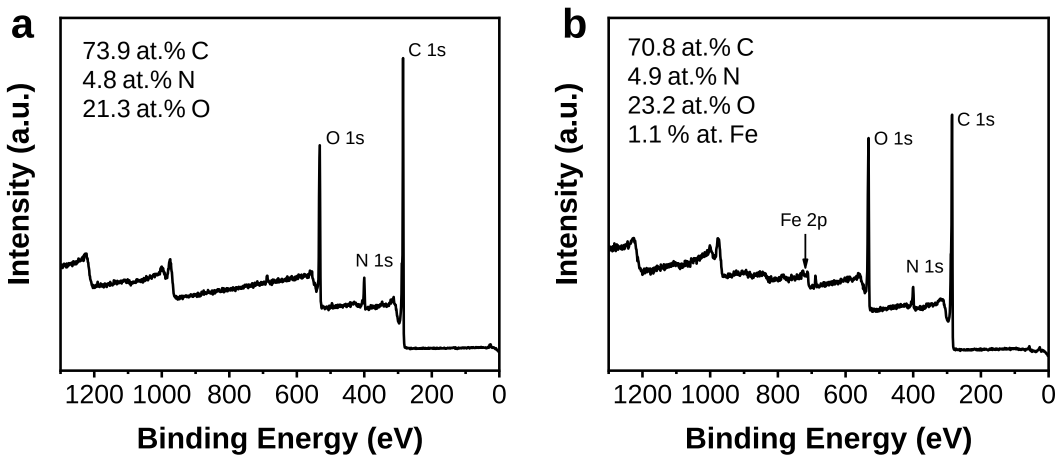

Figure S1. XPS survey spectra of (a) the NGA support and (b) the Fe-NGA catalyst, along with the atomic contents.

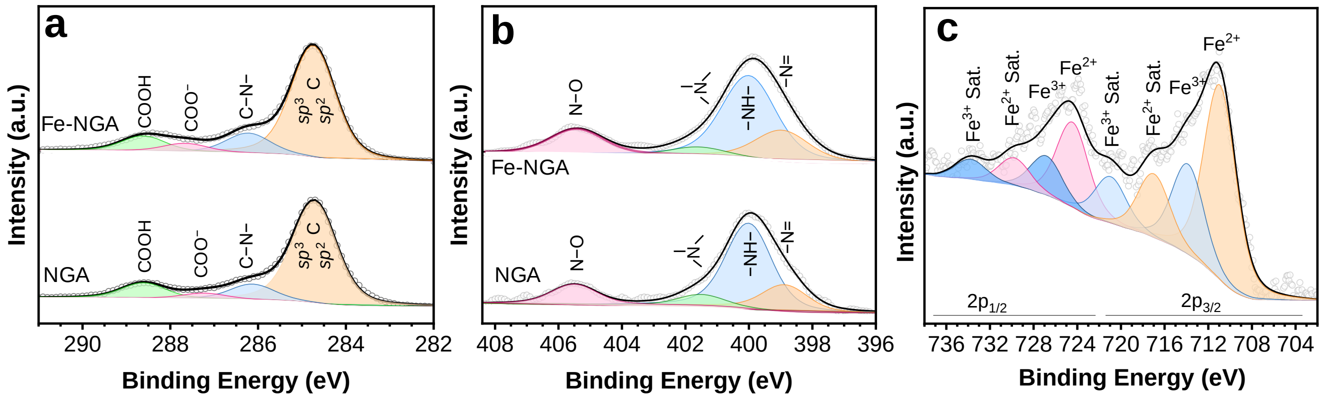

Figure S2. HR-XPS of the NGA support and Fe-NGA catalyst for the spectral regions of: (a) C 1s, (b) N 1s, and (c) Fe 2p. For the C1s region, the protonated and deprotonated carboxylic groups are separated with about 1 eV, as previously known from relevant studies.[1] For the nitrogen analysis, the −N= (sp^2), and −NH− (sp^3) components represent pyridinic (1 part according to the area), piperidinic-like (or pyrrolic; 3 parts), the nitrogen with the three bonds represents graphitic (very low content ~0.3 parts), and N−O is nitro-groups, generated upon HNO_3 treatment of the NG and oxidation of some nitrogen groups.

Table S1. Deconvoluted area % of C 1s region for different binding states.

**B.E**	284.7	286.1	287.2	288.6
**Type**	sp^2	C-N	COO^-	COOH
**NGA**	73.6	11.2	4.1	11.1
**Fe-NGA**	72.0	12.8	5.5	9.7

Table S2. Deconvoluted area % of N 1s region for different binding states.

**B.E**	398.9	400.0	401.5	405.5
**Type**	Pyridinic	Pyrrolic	Graphitic	N-O functionalities
**NGA**	18.7	59.7	7.7	13.9
**Fe-NGA**	20.9	56.5	5.6	17.0

Table S3. Deconvoluted area % of O 1s region for different binding states.

**B.E**	530.1	532	~534.4
**Type**	Carbonyl	Hydroxyl	Na KLL Auger
**NGA**	53.3	44.7	2
**Fe-NGA**	63.4	34.1	2.5

2.2. FTIR analysis

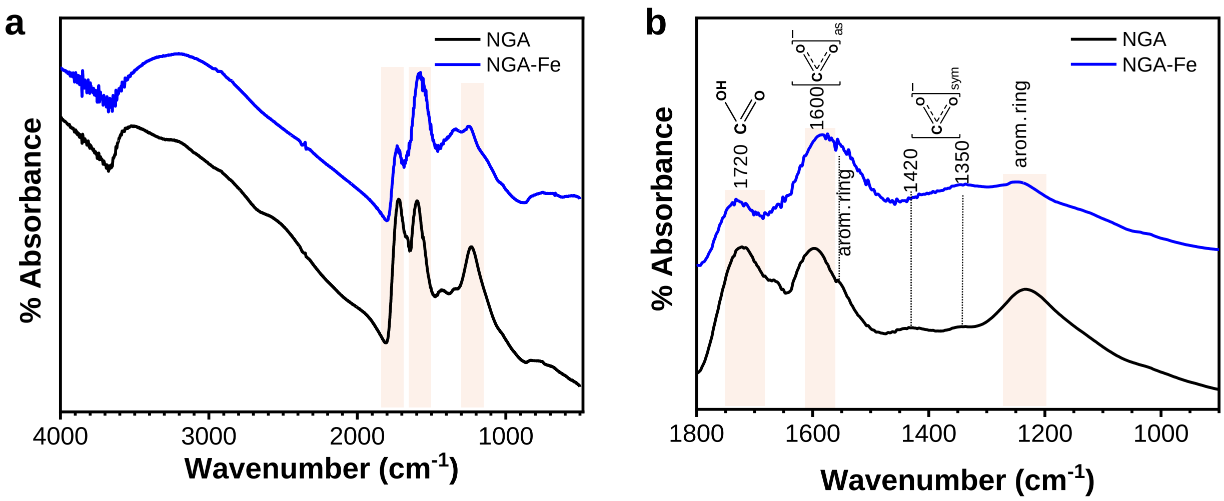

Figure S3. FT-IR spectra of NGA and Fe-NGA. (a) Full-range spectra and (b) enlarged view of the 900–1800 cm^{-1} region.

2.3. XAS analysis

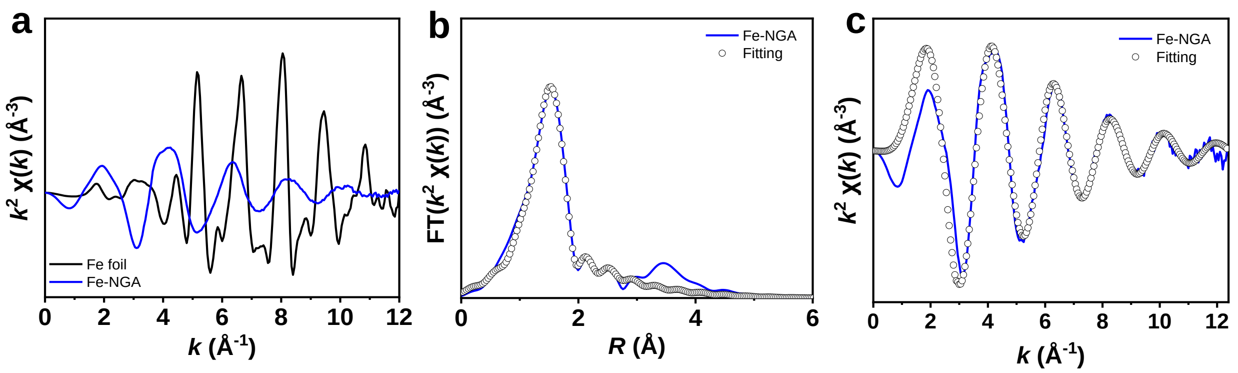

Figure S4. (a) k-space FT-EXAFS spectra of Fe-NGA and Fe foil, (b) R-space fitting for Fe-NGA, and (c) k-space fitting for Fe-NGA.

Table S4. Structural parameters for Fe-NGA catalyst

**Catalyst**	**Shell**	**N^a**	**R^b (Å)**	**$\sigma^{2c}\times10^3$ (Å^2)**	**$\Delta E_0{}^d$ (eV)**	**R factor^e**
Fe-NGA	Fe-N/O	6.05 ± 0.3	2.02	3.8	-1.00 ± 0.86	0.018
	Fe-Fe	1.24 ± 0.3	2.86	4.5		

[a]Coordination numbers, [b]Bond distance, [c]Debye-Waller factors, [d]Inner potential correction, [e]Goodness of fit. $S_0{}^2$ was set to 0.93.
The data range used for data fitting in k-space (Δk) and R-space (ΔR) are 3.0-11.3 Å^{-1} and 1.0-3.0 Å, respectively.

2.4. Modelling and catalyst structure

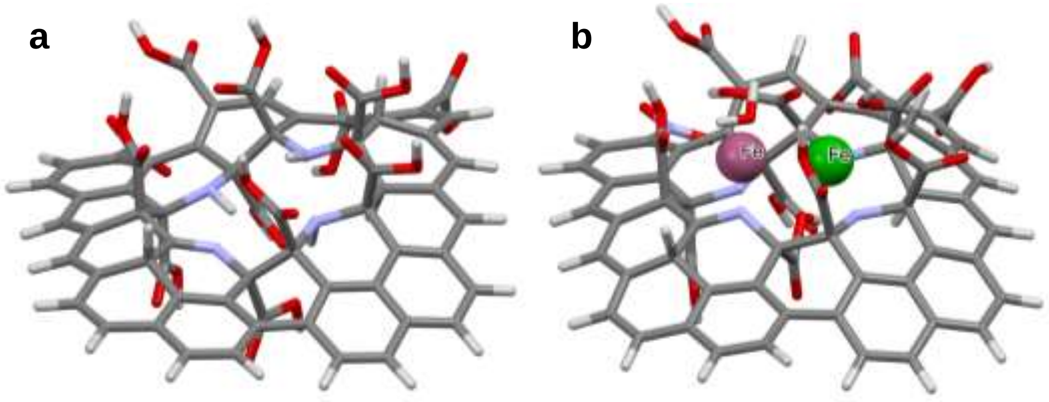

Figure S5. The geometry optimized structures of (**a**) the NGA support (RHF/PM3) and (**b**) the Fe-NGA catalyst (UHF/PM3tm).[2, 3] NGA support: charge = 0, multiplicity = 1, Heat of formation = -2349.23 kJ/mol, E_{HOMO} = -8.72 eV, E_{LUMO} = -2.09 eV, dipole moment = 8.13 debye. Fe-NGA: charge = +2, multiplicity = 6, Heat of formation = -4115.21 kJ mol^{-1}, E_{HOMO} = -13.30 eV, E_{LUMO} = -7.23 eV, dipole moment = 15.42 debye. Cartesian coordinates (.xyz) for NGA are given in Table S7 and for Fe-NGA in Table S8. The theoretical calculations were carried out by the computational software Spartan 10 (v. 1.1.0), Wavefunction Inc., Irvine CA. 92612, USA.

3. Reaction optimization

Table S5. Reaction optimization studies.

No.	Catalyst	HMF Conv. (%)	DFF Sel. (%)	DFF Yield (%)[#]
1	$Fe(NO_3)_3$	23	20	4.6
2	$FeCl_2$	87	19	16.5
3	$Fe(NO_3)_3 + FeCl_2$[b]	30	62	18.6
4	Fe-NGA[a]	100	93	93
5	Fe-NGA[c]	97	95	92.2
6	Fe-NGA[d]	10	6	0.6
7	Fe-NGA[e]	89	95	84.6
8	Fe-NGA[f]	80	91	72.8

Reaction Conditions: [a] 0.125 mmol HMF, 0.25 mmol K_2CO_3, HMF/metal molar ratio in the catalyst was 56:1, 1 MPa O_2, 100 °C, [b] Fe^{2+}:Fe^{3+} = 1, [c] 0.5 MPa O_2, [d] 1 MPa N_2, [e] 0.125 mmol K_2CO_3, [f] 80 °C. [#]Calculated GC yield

The base was crucial for maximizing the catalytic activity (Table S5, entries 3 and 7). Without the addition of base, negligible HMF conversion was observed. Under nitrogen atmosphere negligible activity was recorded.

Solvent effects were examined by testing reactions in isopentanol and acetonitrile, which led to lower HMF conversion and DFF selectivity compared to water. This is attributed to poor solubility of the inorganic base in organic solvents, which hinders formation of the active Fe^{3+}-μ-OH-Fe^{2+} dimer, essential for oxygen activation and efficient catalysis. It is also related to poorer solubility of oxygen itself, which is key for the activation of the catalyst.

To assess the generality of the Fe–NGA catalytic system, we performed oxidation tests with representative benzylic alcohols (e.g., benzyl alcohol and 4-methylbenzyl alcohol) in acetonitrile under otherwise identical conditions. No detectable conversion was observed, confirming that the catalyst is inactive in nonaqueous media. This behavior is attributed to the essential role of OH^- in forming the Fe^{3+}-μ-OH-Fe^{2+} species and to the low proton diffusion in non-protic solvents involved in the key proton-coupled electron transfer steps during O_2 activation.

4. Calibration curves and chromatogram for HMF oxidation to DFF

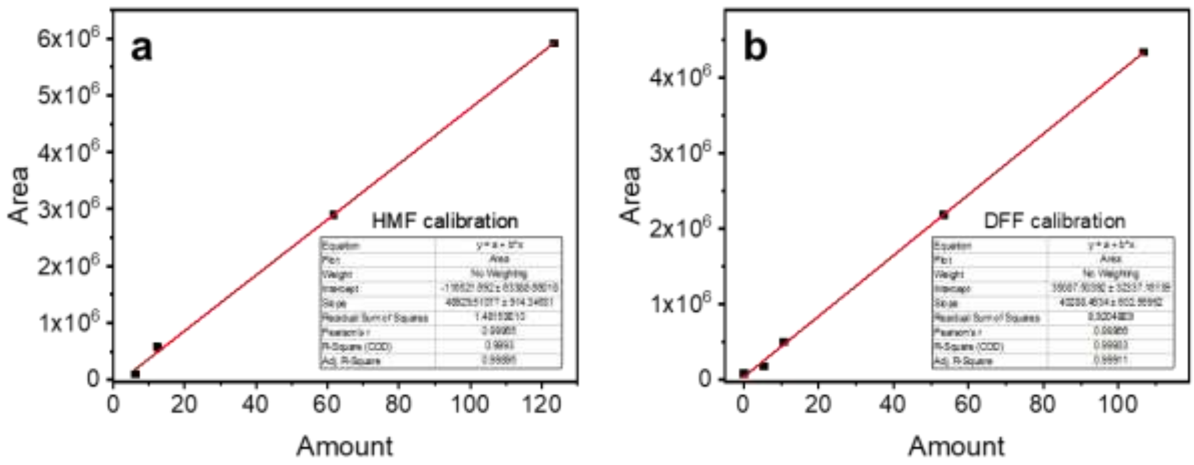

Figure S6. (a) Calibration curve for HMF and (b) for DFF.

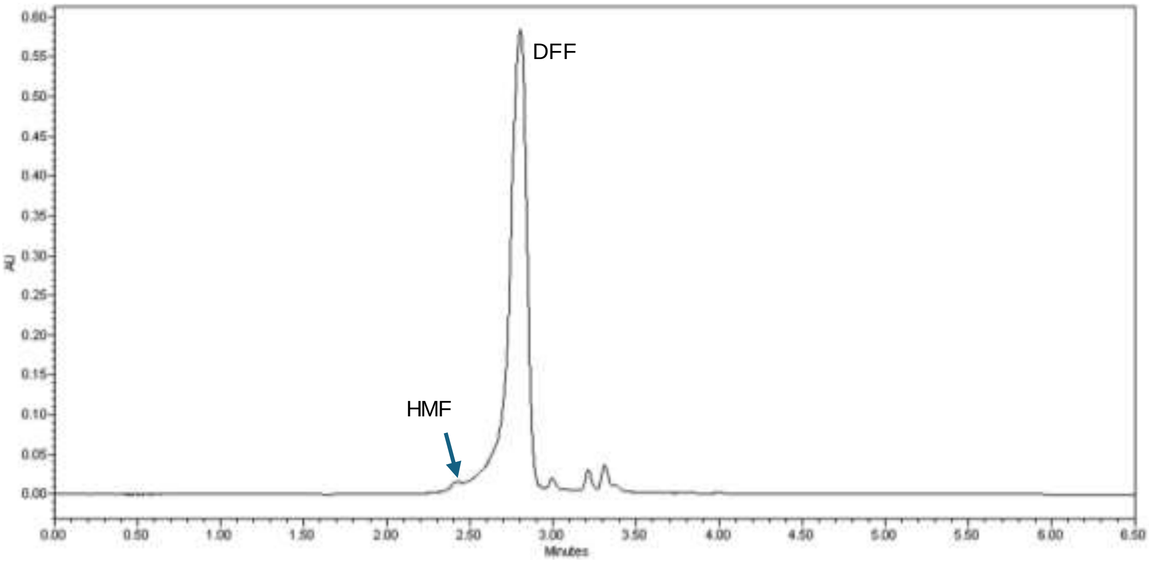

Figure S7. Representative chromatogram of product analysis for the HMF oxidation to DFF.

5. Characterization of the reused catalyst

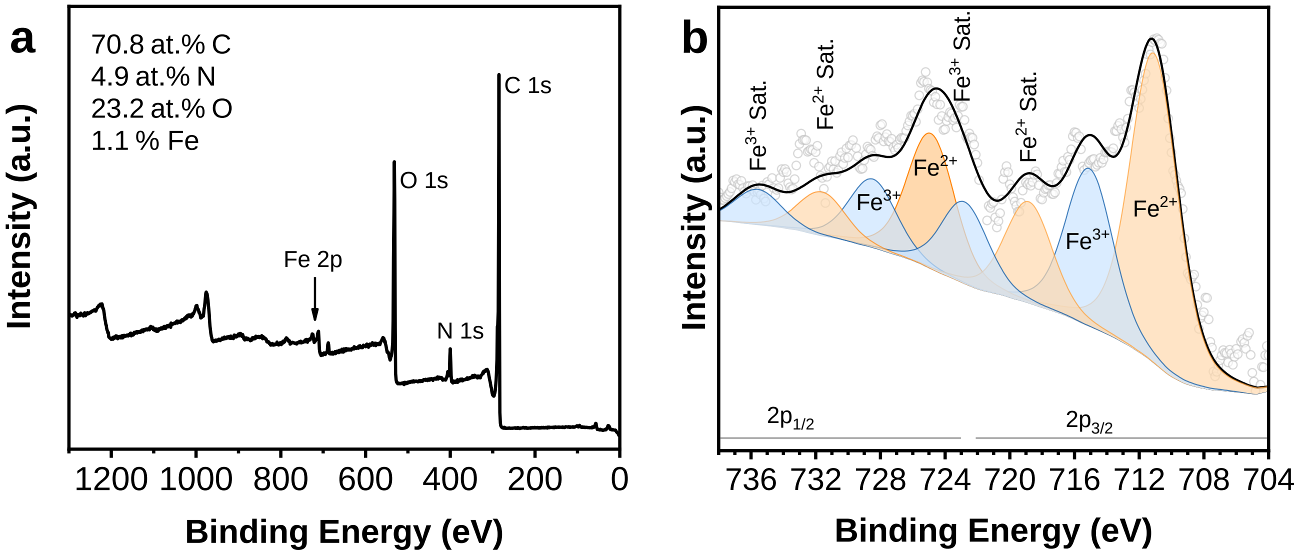

Figure S8. XPS analysis of the reused Fe-NGA catalyst: (a) Survey spectra and (b) HR-XPS of the Fe 2p region.

6. Comparison of performance of Fe-NGA with reported catalysts for HMF to DFF transformation

Table S6. Comparative overview of the Fe-NGA with previously reported catalysts for HMF oxidation.

No.	Catalyst	Reaction conditions	Solvent	HMF Conv. (%)	DFF Sel. (%)	TOF (h^{-1})	Sp. Productivity ($mmol_{DFF}\ g_{cat}^{-1}\ h^{-1}$)	Ref.
1	Cu SAs/p-CNS	0.05 mmol HMF, 0.1 MPa O_2, 24 h, RT, 20W Blue LED	DMF	77.1	85.6	1.2	0.3	4
2	Ru complex@CdS	0.01 mmol HMF, Ar, 16 h, RT, 300 W Xe Lamp	ACN	81	91.8	0.03	0.05	5
3	Oxovanadium complex	2 mmol HMF, 0.4 MPa O_2, 80 °C, 2 h	ACN	99	97.1	9.6	–	6
4	HCP-OH-C-VC	1 mmol HMF, 0.5 MPa O_2, 100 °C, 4 h	ACN	99	99	5.7	6.1	7
5	$ZnIn_2S_4$	10 mmol HMF, AM 1.5G, 2.5 h, RT, light	1M KOH/DMF	~40	>97	-	1.6	8
6	$ZnIn_2S_4/CeO_2$	8 mmol HMF, Air, 1.3 h, RT, Light	ACN	100	96.1	2.9	0.95	9
7	Mn_5O_8	0.5 mmol HMF, 0.5 MPa O_2, 140 °C, 2 h	EtOH	51	94	0.9	2.4	10
8	PdNi@MnO_2	0.32 mmol HMF, 1.5 MPa O_2, 120 °C,1 h	Dioxane	100	99	0.8	6.3	11
9	FeCo/C	1 mmol HMF, 1 MPa O_2, 100 °C, 6 h	Toluene	100	99	0.8	0.4	12
10	Ru/γ-Al_2O_3	3.96 mmol HMF, 0.5 MPa O_2, 110 °C, 5 h	DMF	91.2	21.2	0.01	0.3	13
11	Au NPs@sPSB	0.13 mmol HMF, 1.5 MPa O_2, 80 °C, 16 h	DMF/DMA	78	80	0.98	0.1	14
12	Ru@CTF	1 mmol HMF, 2 MPa air, 80 °C, 3 h	MTBE[a]	97.3	72.7	9.4	3.5	15
13	Fe_2O_3@HAP-Ru	0.79 mmol HMF, 20 mL min^{-1} O_2, 80 °C, 4 h	Toluene	100	89.1	5.9	1.2	16
14	$Cu(NO_3)_2$ + $VOSO_4$	10 mmol HMF, 0.1 MPa O_2, 80 °C, 5 h	ACN	99	99	4.9	–	17
15	$Mn_6Fe_1O_x$	1 mmol HMF, 1.5 MPa O_2, 110 °C, 5 h	DMF/H_2O	97	98	0.2	2.4	18
16	Ru/$MnCo_2O_4$	2 mmol HMF, 1 MPa O_2, 130 °C, 3 h	Toluene	98.3	100	0.5	3.3	19
**17**	**Fe-NGA**	**0.1 mmol HMF, 1 MPa O_2, 100 °C, 3 h**	**H_2O**	**100**	**93**	**17.4**	**12.5**	**This Work**
**18**	**Fe-NGA**	**0.1 mmol HMF, 0.5 MPa O_2, 100 °C, 3 h**	**H_2O**	**97**	**95**	**~17.3**		

[a]MTBE = methyl t-butyl ether

Previous attempts to perform the selective oxidation of HMF to DFF in pure water have resulted to extremely low specific productivity (0.2 - 0.23 $mmol_{DFF}$ g_{cat}^{-1} h^{-1}) and catalyst instability,[20] a photocatalyst based on CdS gave a 0.1 $mmol_{DFF}$ g_{cat}^{-1} h^{-1},[21] or another photocatalyst based on $Cu_2O\|TiO_2$ delivered 0.6 $mmol_{DFF}$ g_{cat}^{-1} h^{-1} and very low yield of 23%.

7. Reaction mechanism and computational models

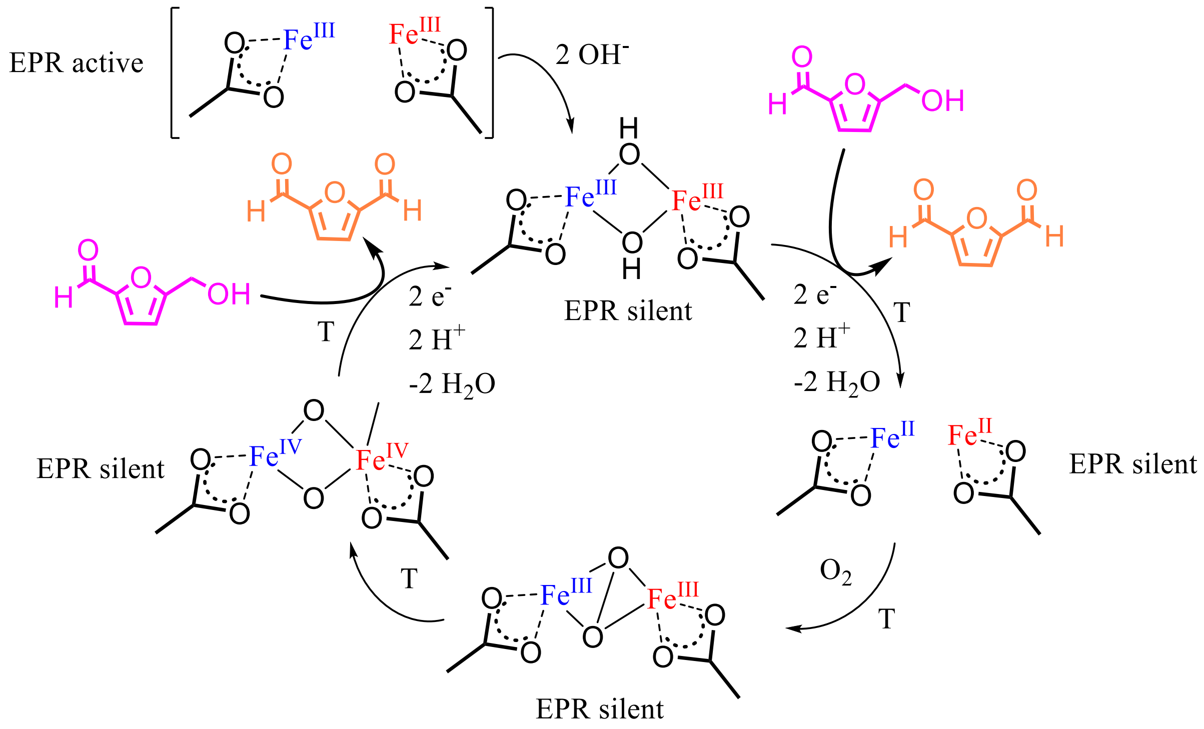

Figure S9. Other possible reaction mechanism for HMF oxidation by the Fe-NGA catalyst involving Fe(III)-Fe(III) and Fe(II)-Fe(II) pairs.

Comments: The other possible pathway available in Fe-NGA, as shown in Figure 4 of the main manuscript, can also contribute to catalysis, and employ a combination of redox-active Fe^{3+}–Fe^{3+} and/or Fe^{2+}–Fe^{2+} dimers. However, during catalytic oxidation of HMF, none of the intermediates shown above provide access to the mixed valent states (Fe^{3+}–Fe^{2+}; Fe^{3+}–Fe^{4+}), thus no catalytic intermediates reported in the reaction scheme above can give rise to the experimentally observed EPR signal shown in Figure 2d,k,g,n.

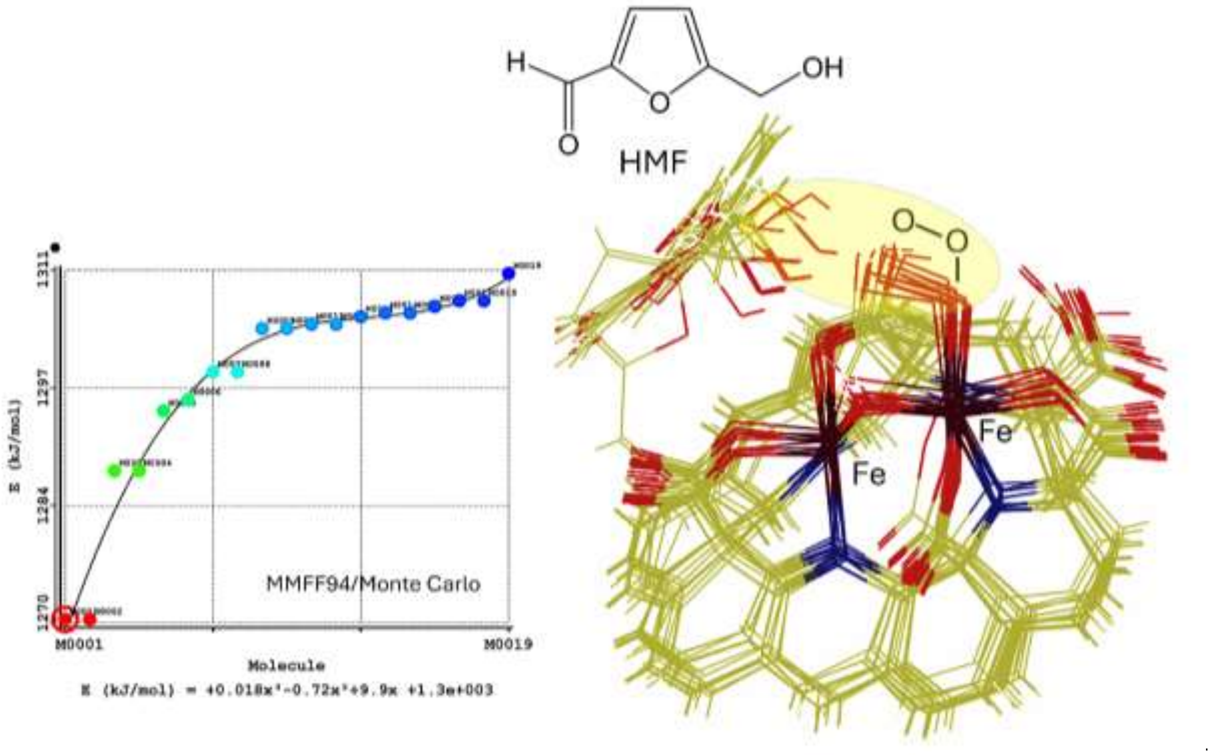

Figure S10. The interaction of HMF with Fe-NGA when intermediate [II] is present in the reaction environment, Fe(III)-μ-OH-(O_2)-Fe(IV). The alcohol chain (–CH_2–OH) closely interacts with the Fe(IV)-O_2^- center (highlighted by a yellow oval), while the formyl part of HMF consistently positioned itself over the Fe(III) center. Calculation of the conformer's distribution shown in the figure employed combination of MMFF94[22] and Monte Carlo algorithm, which uses a simulated annealing to generate a set of possible conformations of the two molecules, HMF and Fe-NGA. The initial temperature for the Monte Carlo (MC)/Simulated-Annealing algorithm was set to $T = 5000$ K. Restricted searches were applied, using an energy window of $E_{max} = 40$ kJ mol^{-1} from a pool of 1000 conformers. The best scored conformers (19) are shown as overlayed structures on the right and their energy difference on the left-plot.

8. Cartesian coordinates of computational models

Table S7. Cartesian coordinates for the NGA system obtained from geometry optimization shown in Figure S5a.

```
  Run type: Geometry optimization
            (Analytical Gradient)
            (MM/Amide correction used)
  Model: RHF/PM3
  Number of shells: 217
   125 S shells
    92 P shells
  Number of basis functions:  401
  Number of electrons:  454
  Use of molecular symmetry disabled
  Molecular charge:      0
  Spin multiplicity:     1

125
M0001
C       6.229008    0.082303   -3.528431
C       5.621701    2.231156   -2.541266
C       4.347896    0.171343   -1.877699
C       4.469273    1.661709   -1.978215
C       5.022721   -0.543898   -3.000827
C       6.527694    1.371268   -3.286823
H       4.920165   -0.110858   -0.945502
H       6.887261   -0.542273   -4.144125
H       7.445905    1.824301   -3.678485
C       4.574517   -1.728856   -3.458882
H       5.123798   -2.253429   -4.252562
C       3.352733   -2.361320   -2.989242
C       2.601883   -1.771124   -1.812573
C       2.936699   -0.283838   -1.635460
C       2.879993   -3.464681   -3.609465
H       3.437152   -3.924848   -4.436333
C       1.618044   -4.088601   -3.239159
C       0.727984   -3.429329   -2.377803
C       1.048031   -1.992572   -2.041319
C       1.263586   -5.328420   -3.775725
H       1.972612   -5.884664   -4.400117
C      -0.016299   -5.812575   -3.565893
H      -0.324116   -6.735576   -4.078698
C      -0.913341   -5.138905   -2.725341
C      -0.483402   -4.024333   -1.972673
N       0.360993   -1.546337   -0.810418
C      -1.227312   -3.454234   -0.836116
C      -1.029143   -1.967744   -0.518607
H       0.523088   -0.546521   -0.699086
C      -2.046154   -4.140845    0.007329
C      -3.003645   -3.403654    0.839192
H      -3.953594   -3.919086    1.039181
C      -2.715827   -2.155435    1.272766
C      -1.275383   -1.755603    1.064169
N      -0.983192   -0.352163    1.430190
C      -1.645422    0.354213    2.566830
C      -3.081949   -0.207909    2.864192
H      -3.019495   -0.797758    3.824780
```

C	-3.653074	-1.246904	1.924166
N	1.998819	0.501859	-1.214287
C	2.140916	1.959652	-1.059870
C	3.499292	2.493243	-1.427135
C	3.754438	3.857947	-1.218482
C	4.934700	4.406400	-1.722864
H	5.118999	5.481314	-1.581617
C	5.839909	3.606671	-2.413464
H	6.739292	4.054778	-2.851521
C	1.852608	2.463333	0.408536
C	1.814164	3.975434	0.309214
C	2.763566	4.634009	-0.459278
H	0.011897	-0.229396	1.504480
N	0.576325	1.870511	0.903539
C	-0.158985	2.562804	2.011020
C	-0.264437	4.045363	1.694620
C	0.770709	4.696070	0.953335
H	-0.054392	1.798026	0.112223
C	0.731954	6.099891	0.807692
C	1.758939	6.762304	0.084110
H	1.738194	7.856509	0.018810
C	2.749824	6.048202	-0.532800
H	3.552526	6.542224	-1.100145
C	-0.344698	6.835942	1.375944
H	-0.353873	7.926864	1.266978
C	-1.353383	6.192257	2.033702
H	-2.194525	6.753616	2.456811
C	-1.320459	4.780050	2.191614
C	-1.586679	1.876582	2.109512
H	-1.995059	1.870940	1.057201
C	-2.550266	2.763190	2.831055
C	-2.413021	4.106708	2.866531
H	-3.159095	4.737110	3.368250
C	-3.787800	2.177075	3.297868
H	-4.552109	2.868749	3.680770
C	-4.067351	0.867341	3.189926
C	-5.006860	-1.377125	1.824429
C	-5.894796	-0.634648	2.713910
C	-5.434599	0.423104	3.408936
H	-6.069030	0.996175	4.096462
C	3.123148	-2.502046	-0.551482
O	3.953572	-2.099304	0.242401
O	2.679839	-3.759372	-0.347735
H	2.924319	-4.045954	0.527511
C	0.606982	-1.195709	-3.316485
O	1.238642	-0.426671	-4.014002
O	-0.657133	-1.416058	-3.743866
H	-0.831705	-0.879960	-4.512336
C	-2.057653	-1.095966	-1.281746
O	-2.037424	0.109898	-1.454957
O	-3.091937	-1.751222	-1.840180
H	-3.692003	-1.137938	-2.253078
C	-0.345483	-2.681493	1.903660
O	0.847169	-2.879662	1.792863
O	-0.941099	-3.324497	2.936356
H	-0.277802	-3.751642	3.470180
C	-0.913870	0.088117	3.908206
O	-0.144260	-0.816280	4.174206
O	-1.233796	0.885377	4.954576
H	-0.731115	0.614945	5.718116
C	0.631206	2.465177	3.333483

O	1.433578	1.625610	3.700133
O	0.430557	3.453055	4.234554
H	0.988328	3.297861	4.991476
C	2.999707	2.036444	1.357905
O	3.722828	2.756512	2.022325
O	3.229024	0.712176	1.437011
H	3.855862	0.521900	2.128114
C	1.089835	2.517912	-2.064140
O	1.262257	2.985572	-3.169815
O	-0.193762	2.438403	-1.639909
H	-0.799974	2.681758	-2.332114
C	-5.706188	-2.261493	0.846423
O	-6.334276	-3.284375	1.052829
O	-5.686026	-1.812095	-0.429186
H	-6.148922	-2.423761	-0.994684
C	-7.310254	-1.054656	2.919613
O	-7.809784	-1.527719	3.922214
O	-8.140824	-0.820455	1.876194
H	-9.011788	-1.150691	2.074545
C	-2.101282	-5.613227	0.217812
O	-3.076147	-6.315922	0.405227
O	-0.895697	-6.227202	0.295495
H	-1.019672	-7.155938	0.467887
N	-2.342495	-5.610908	-2.812555
O	-2.567729	-6.803864	-2.776211
O	-3.223802	-4.797717	-2.988800

Table S8. Cartesian coordinates for the Fe-NGA system obtained from geometry optimization shown in Figure S5b.

```
M0001
Run type: Geometry optimization
(Analytical Gradient)
(MM/Amide correction used)
Model: UHF/PM3D
(PM3 H...H correction used)
Number of shells: 224
127 S shells
95 P shells
2 5D shells
Number of basis functions: 422
Number of electrons: 473
Use of molecular symmetry disabled
Molecular charge: 2
Spin multiplicity: 6

127
M0001
C       5.861635    0.236228   -3.828434
C       5.397217    2.261582   -2.532089
C       4.355472    0.138769   -1.846713
C       4.341789    1.632178   -1.822942
C       4.870599   -0.479625   -3.100816
C       6.156355    1.545922   -3.512922
H       5.136951   -0.164130   -1.082297
H       6.383566   -0.265225   -4.654004
H       6.954859    2.077315   -4.047608
C       4.462189   -1.740895   -3.500354
H       4.967838   -2.216873   -4.354052
C       3.348500   -2.421170   -2.927276
C       2.627557   -1.863553   -1.726437
C       3.104876   -0.438085   -1.302239
C       2.838203   -3.530695   -3.558725
H       3.372408   -3.990740   -4.402455
C       1.537028   -4.092509   -3.202174
C       0.666373   -3.353892   -2.387469
C       1.053703   -1.924018   -2.094716
C       1.130410   -5.320472   -3.721295
H       1.818458   -5.926455   -4.326257
C      -0.188066   -5.735832   -3.531893
H      -0.533250   -6.641516   -4.059314
C      -1.060197   -5.016776   -2.708452
C      -0.581265   -3.894418   -1.982396
N       0.248952   -1.301427   -1.014699
C      -1.236677   -3.305004   -0.827844
C      -1.096365   -1.812799   -0.621008
C      -1.955586   -4.045770    0.161076
C      -2.717263   -3.434014    1.130980
H      -3.288114   -4.026715    1.863079
C      -2.678571   -1.984496    1.312685
C      -1.340557   -1.434459    0.948573
N      -1.255248    0.021285    1.177545
C      -2.054075    0.727372    2.239272
C      -3.491927    0.171046    2.345497
H      -3.689010   -0.129755    3.418460
C      -3.730927   -1.163439    1.667281
```

N	2.440099	0.181829	-0.323963
C	2.257344	1.707865	-0.346534
C	3.434174	2.371826	-1.038319
C	3.701241	3.757772	-0.867026
C	4.893496	4.314827	-1.381282
H	5.206998	5.327000	-1.094382
C	5.702986	3.605996	-2.250511
H	6.580891	4.078802	-2.710435
C	1.851953	2.620394	0.954430
C	1.811201	4.099726	0.669232
C	2.761123	4.643099	-0.199363
N	0.447288	2.269607	1.239208
C	-0.502426	2.957454	2.121200
C	-0.473755	4.393650	1.715402
C	0.701903	4.910002	1.100309
C	0.696074	6.302790	0.801716
C	1.756023	6.852457	0.041199
H	1.766774	7.930991	-0.165795
C	2.731168	6.031589	-0.490457
H	3.454937	6.481089	-1.181158
C	-0.404254	7.117989	1.193840
H	-0.343351	8.201205	1.024155
C	-1.547977	6.568186	1.738351
H	-2.406349	7.200729	1.997243
C	-1.623757	5.166122	1.949755
C	-1.888470	2.255668	1.833883
H	-2.001213	2.278666	0.710481
C	-3.014398	3.134359	2.270309
C	-2.841238	4.525342	2.343411
H	-3.687059	5.159078	2.644443
C	-4.301519	2.573059	2.348212
H	-5.159018	3.243267	2.510275
C	-4.553403	1.200586	2.152135
C	-5.112891	-1.538205	1.509733
C	-6.133484	-0.597265	1.663222
C	-5.862976	0.762363	1.913355
H	-6.690374	1.486292	1.908979
C	2.983977	-2.750695	-0.490337
O	3.872848	-2.502677	0.320016
O	2.166237	-3.773857	-0.113463
C	0.728997	-1.104672	-3.378709
O	-0.181365	-1.309434	-4.152669
O	1.515825	-0.036490	-3.626053
H	1.204239	0.434836	-4.394914
C	-2.134782	-1.016903	-1.457590
O	-2.197728	0.207063	-1.502757
O	-3.004073	-1.725485	-2.171259
H	-3.595885	-1.166982	-2.669095
C	-0.113122	-2.019055	1.686299
O	0.524621	-1.437126	2.613024
O	0.753721	-2.990175	1.242437
C	-1.399853	0.523231	3.642991
O	-1.818338	0.559983	4.763131
O	-0.021788	0.182540	3.624437
C	-0.078174	2.925233	3.597178
O	-0.475580	3.542338	4.550664
O	0.969776	2.064334	3.891871
C	2.733737	2.076526	2.063844
O	3.659359	2.435182	2.730396
C	0.866542	1.645177	-1.002573
O	0.383179	2.176675	-1.963671

O	0.109552	0.761361	-0.254258
H	-0.860270	0.759483	-0.494743
C	-5.511167	-2.923819	1.101661
O	-5.789074	-3.852484	1.835360
O	-5.570936	-3.096952	-0.233199
H	-5.885345	-3.968615	-0.457548
C	-7.575541	-0.970149	1.497313
O	-8.199775	-1.059045	0.460790
O	-8.211141	-1.193868	2.666802
H	-9.121279	-1.433058	2.515657
C	-1.822731	-5.530040	0.296939
O	-2.667144	-6.390995	0.218711
O	-0.558065	-5.916481	0.627636
H	-0.525641	-6.860948	0.756280
N	-2.530944	-5.427226	-2.766754
O	-2.785960	-6.576332	-3.039092
O	-3.379427	-4.589031	-2.565088
O	2.334991	0.738200	2.260857
H	1.420088	2.360683	4.682179
H	2.649358	-4.398235	0.426546
H	2.656938	0.332921	3.072949
H	0.462848	0.461163	4.414352
H	0.418385	-3.630266	0.593114
Fe	1.455490	-1.251261	0.379180
Fe	0.412460	0.556269	1.777453
H	2.261352	-1.870403	2.700344
O	2.596765	-1.216875	2.062088
H	3.488244	-1.486714	1.768449

Table S9. Cartesian coordinates for the Fe-NGA system (Model I) obtained from single point UHF/PM6 shown in Figure 4b, main text.

```
89
M0001
C      -3.670381    2.431756   -0.359434
C      -3.864047    3.797605    0.236444
C      -3.374981    4.180189    1.429106
C      -2.540581    3.337562    2.271081
C      -2.100309    1.972019    1.750366
C      -2.404144    1.836977    0.196463
C      -2.066815    3.757691    3.465308
C      -0.255272    1.926558    3.603538
C      -0.562861    1.754131    2.135379
C       0.770412    1.284713    4.201567
N      -0.044704    0.404472    1.674170
C       1.679294    0.391268    3.492159
C       1.418785    0.068842    1.997101
C       2.802560   -0.103758    4.067654
C       3.730867   -0.954721    3.320119
C       3.347091   -1.625519    2.208929
C       1.908563   -1.459810    1.707702
N       1.881649   -2.017401    0.350342
C       2.779336   -2.324816   -0.722607
C       4.189744   -1.993535   -0.210296
N      -1.849656    0.818458   -0.454299
C      -1.737010    0.555714   -1.953063
C      -3.133533    1.022959   -2.479199
C      -3.712418    0.363554   -3.508745
C      -1.504747   -1.104862   -2.343616
C      -1.805406   -1.158773   -3.844327
C      -2.926080   -0.567132   -4.320154
N      -0.187047   -1.895020   -2.140316
C       0.922304   -2.190024   -2.982218
C       0.575069   -1.655902   -4.350724
C      -0.674884   -1.358952   -4.759031
C       2.323054   -1.722350   -2.211321
C      -2.912664    0.882175    2.467799
O      -3.698642    1.090902    3.401217
O      -2.735085   -0.380359    2.005868
C       0.866918   -2.360054    2.476570
O      -0.011532   -3.043740    1.824331
O       0.975528   -2.567148    3.810996
C       2.658743   -3.867330   -0.876850
O       3.560036   -4.628448   -1.227993
O       1.410677   -4.317009   -0.525068
C       1.049887   -3.711296   -3.340169
O       0.278844   -4.642434   -3.144771
O       2.212027   -4.008694   -3.989851
C      -2.607757   -1.849562   -1.456035
O      -3.554716   -2.507285   -1.913862
O      -2.396942   -1.852980   -0.077840
H      -3.589616    5.183629    1.786436
H      -2.356685    4.729155    3.854077
H       0.983707    1.488867    5.246233
```

H	2.107813	-4.973810	-4.096427
H	3.151177	-1.968500	-2.890586
H	2.314691	-0.629779	-2.165380
H	4.949627	-2.543672	-0.778163
H	4.764031	-0.970151	3.657490
H	3.083814	0.165796	5.081826
H	-4.494160	1.799952	0.002311
H	-4.478425	4.492765	-0.327165
H	4.424570	-0.934993	-0.350696
H	1.379741	-1.637676	-5.077450
H	-0.823593	-1.069491	-5.793569
H	-3.107117	-0.504229	-5.388178
H	-4.656606	0.695147	-3.924240
C	-1.093984	2.968752	4.328586
C	4.326277	-2.354939	1.298876
C	-3.675970	2.329559	-1.895885
H	-1.662314	2.488815	5.134040
H	5.356597	-2.138360	1.606952
H	4.183073	-3.434119	1.433255
H	-4.700096	2.497935	-2.254252
H	-3.080360	3.153347	-2.310927
H	-0.411476	3.692013	4.793108
Fe	-1.372290	-0.533892	0.708205
Fe	0.122374	-2.743598	-0.115658
H	1.688539	-2.014950	4.163026
O	0.109474	-0.414408	-0.434550
H	0.654582	0.145215	-1.021691
C	0.167507	2.871664	1.339887
O	0.433635	4.014618	1.683698
O	0.435513	2.372871	0.111480
H	0.114884	1.400853	0.192085
C	2.474163	0.995363	1.257390
O	3.098968	1.931928	1.750975
O	2.687078	0.624257	-0.021566
H	2.115222	-0.187268	-0.158846
C	-0.801896	1.519142	-2.801295
O	-0.549728	1.477858	-4.003079
O	-0.440095	2.639037	-2.127826
H	-0.585455	2.421717	-1.176602

Table S10. Cartesian coordinates for the Fe-NGA system (Model II) obtained from single point UHF/PM6 shown in Figure 4b, main text.

91
M0001

C	3.652444	0.411629	-2.758194
C	3.833137	-0.249055	-4.091230
C	3.317945	-1.436913	-4.434570
C	2.473261	-2.228071	-3.562729
C	2.149045	-1.725850	-2.136763
C	2.548306	-0.215057	-1.943187
C	1.904147	-3.373759	-4.000250
C	0.251970	-3.478728	-2.042049
C	0.588392	-1.988158	-1.888838
C	-0.666705	-4.105589	-1.272944
N	0.157105	-1.645906	-0.474916
C	-1.495626	-3.427109	-0.278602
C	-1.284581	-1.897543	-0.029073
C	-2.486144	-4.070224	0.388920
C	-3.432459	-3.328921	1.225188
C	-3.099176	-2.136581	1.770188
C	-1.660897	-1.642525	1.609219
N	-1.593867	-0.243797	2.258031
C	-2.578182	0.856627	2.251903
C	-3.869707	0.252944	1.639916
N	2.182127	0.419768	-0.829000
C	1.892116	1.873339	-0.699635
C	3.019806	2.561862	-1.493017
C	3.518667	3.742655	-1.075258
C	1.777082	2.403820	0.846284
C	1.866101	3.952277	0.725410
C	2.787630	4.518056	-0.087067
N	0.567212	2.132968	1.730714
C	-0.613767	2.844860	2.029822
C	-0.393130	4.281886	1.649521
C	0.753197	4.784233	1.166050
C	-1.927377	2.111295	1.463789
C	2.988095	-2.608736	-1.191802
O	3.722759	-3.536936	-1.568631
O	2.924164	-2.349327	0.130484
C	-0.679882	-2.581301	2.442871
O	-0.899108	-3.745471	2.799096
O	0.486763	-1.990241	2.847353
C	-3.085318	1.442939	3.618023
O	-2.603281	1.349106	4.738221
O	-4.147376	2.277837	3.495824
C	-0.607244	2.780744	3.560755
O	-0.115373	1.776863	4.215716
O	-1.172658	3.809908	4.248736
C	3.063327	1.892193	1.579163
O	3.783198	2.618812	2.286702
O	3.358104	0.559848	1.518551
H	3.514011	-1.823604	-5.429675
H	2.120591	-3.747814	-4.995339
H	-0.869564	-5.157676	-1.435995

H	-1.146295	3.489289	5.170458
H	-2.711375	2.876357	1.378495
H	-1.715785	1.789234	0.440474
H	-4.755653	0.871769	1.819459
H	-4.451587	-3.696167	1.244449
H	-2.684448	-5.122836	0.231471
H	4.580381	0.250892	-2.191320
H	4.450597	0.279579	-4.809531
H	-3.810856	0.202067	0.557194
H	-1.217159	4.962916	1.834672
H	0.804799	5.845958	0.950620
H	2.817426	5.592037	-0.237949
H	4.279041	4.258850	-1.648881
C	0.943681	-4.205960	-3.177508
C	-4.130882	-1.148676	2.240291
C	3.397314	1.923807	-2.822199
H	-4.342171	2.525031	4.419167
H	1.484646	-5.077633	-2.790525
H	-5.133050	-1.471646	1.932381
H	-4.137402	-1.109716	3.335158
H	4.283279	2.419876	-3.239581
H	2.578091	2.120979	-3.527768
H	0.169915	-4.577077	-3.862055
Fe	2.083239	-0.745216	0.690472
Fe	0.398521	0.168831	3.061122
O	0.463129	-0.005524	1.159228
O	-0.295711	-0.565399	4.866429
O	-1.631615	-1.102391	4.668783
H	-1.743251	-0.729074	3.747801
C	0.560617	2.235935	-1.419647
O	0.217007	3.315785	-1.894953
O	-0.193220	1.122776	-1.519780
H	0.365643	0.403711	-1.065018
C	-0.155905	-1.228593	-3.072476
O	0.260928	-0.342835	-3.812502
O	-1.403468	-1.675520	-3.369726
H	-1.699765	-0.870193	-3.838610
C	-2.381086	-1.167532	-0.919880
O	-2.467922	-0.005747	-1.293839
O	-3.417975	-1.938480	-1.368541
H	-3.827963	-1.263501	-1.938824

Table S11. Cartesian coordinates for the Fe-NGA system (**Model III**) obtained from single point UHF/PM6 shown in Figure 4b, main text.

90
M0001

C	3.766834	0.266704	-2.498773
C	4.073132	-0.406223	-3.801857
C	3.550427	-1.576342	-4.196900
C	2.567316	-2.318311	-3.429762
C	2.109247	-1.787529	-2.068632
C	2.527641	-0.299103	-1.862096
C	1.975418	-3.418339	-3.940248
C	0.164145	-3.437512	-2.128423
C	0.532589	-1.961262	-1.945072
C	-0.726898	-4.072214	-1.339237
N	0.107124	-1.583382	-0.575316
C	-1.507783	-3.407945	-0.304114
C	-1.309204	-1.882325	-0.056503
C	-2.446880	-4.077776	0.403453
C	-3.370299	-3.364431	1.273913
C	-3.053360	-2.163172	1.804990
C	-1.628992	-1.596969	1.613531
N	-1.628462	-0.232659	2.293983
C	-2.623007	0.802091	2.353741
C	-3.912420	0.200491	1.741758
N	2.015991	0.346844	-0.821029
C	1.824410	1.784542	-0.644504
C	3.113036	2.408980	-1.253504
C	3.663491	3.502182	-0.686493
C	1.639014	2.205860	0.970630
C	1.844692	3.727361	0.941503
C	2.888996	4.272570	0.275158
N	0.410596	1.846551	1.957070
C	-0.745040	2.746736	2.172811
C	-0.487821	4.100541	1.549838
C	0.694013	4.575482	1.149246
C	-2.090306	2.101714	1.657115
C	2.738310	-2.653142	-0.943491
O	3.547506	-3.569046	-1.142435
O	2.340751	-2.361983	0.330723
C	-0.609779	-2.482992	2.481386
O	-0.700349	-3.697591	2.697237
O	0.450194	-1.807109	3.029268
C	-3.123037	1.134505	3.797436
O	-2.924672	0.550231	4.852936
O	-4.032519	2.145344	3.827333
C	-0.781618	3.243541	3.665788
O	0.140431	3.329329	4.471044
O	-1.947545	3.824483	4.046737
C	2.861226	1.494724	1.648553
O	3.770097	2.068208	2.262979
O	2.839032	0.129491	1.612558
H	3.847593	-1.982945	-5.159244
H	2.281499	-3.804587	-4.907644
H	-0.933682	-5.124122	-1.505724

H	-1.684236	4.095824	4.947994
H	-2.880866	2.863036	1.706016
H	-1.969826	1.892094	0.587194
H	-4.798644	0.802960	1.977005
H	-4.378034	-3.759958	1.334554
H	-2.628994	-5.134616	0.251082
H	4.597548	0.038697	-1.815934
H	4.798716	0.085336	-4.442152
H	-3.872465	0.192152	0.654474
H	-1.332687	4.785236	1.552308
H	0.749713	5.606072	0.813289
H	2.989885	5.349414	0.175670
H	4.542045	3.974475	-1.110403
C	0.848617	-4.166445	-3.266530
C	-4.128540	-1.229238	2.290592
C	3.613191	1.793253	-2.555174
H	-4.185689	2.214155	4.788150
H	1.235519	-5.131709	-2.918607
H	-5.114498	-1.573337	1.953537
H	-4.155974	-1.243405	3.384819
H	4.572243	2.242792	-2.842720
H	2.900156	2.058829	-3.347625
H	0.092176	-4.371966	-4.034867
Fe	1.577514	-0.724654	0.550744
Fe	0.158266	0.045929	3.462630
O	-0.263450	0.613818	5.069267
H	-0.195459	0.702843	1.205755
O	-0.045516	0.087257	0.453758
C	0.646920	2.353457	-1.493725
O	0.572581	3.477635	-1.988280
O	-0.323771	1.443474	-1.704319
H	-0.073491	0.631903	-1.163371
C	-0.111934	-1.142232	-3.117356
O	0.411692	-0.304282	-3.843398
O	-1.404451	-1.441354	-3.394274
H	-1.649777	-0.607674	-3.844761
C	-2.411640	-1.135237	-0.896238
O	-2.461264	0.038680	-1.241130
O	-3.464331	-1.882608	-1.334174
H	-3.907720	-1.198876	-1.869145

Table S12. Cartesian coordinates for the Fe-NGA system (**Model IV**) obtained from single point UHF/PM6 shown in Figure 4b, main text.

```
89
M0001
C       3.730676    0.249599   -2.409209
C       4.028390   -0.451629   -3.702818
C       3.482198   -1.609655   -4.094536
C       2.494430   -2.322872   -3.313985
C       2.093281   -1.799262   -1.915045
C       2.593673   -0.361065   -1.640760
C       1.864125   -3.411562   -3.808110
C       0.017870   -3.321598   -2.028090
C       0.503968   -1.873048   -1.819190
C      -1.065242   -3.831019   -1.401988
N      -0.067540   -1.499329   -0.425100
C      -1.940613   -3.064240   -0.515999
C      -1.579888   -1.573196   -0.141060
C      -3.088079   -3.604852   -0.045900
C      -4.005299   -2.833916    0.775180
C      -3.574136   -1.779948    1.500632
C      -2.080167   -1.398258    1.436357
N      -1.945054   -0.139748    2.181546
C      -2.785799    0.957076    2.408290
C      -4.212848    0.627534    1.947635
N       2.151351    0.269902   -0.555038
C       1.816311    1.715929   -0.492440
C       2.971356    2.402015   -1.268690
C       3.485349    3.579235   -0.863411
C       1.630945    2.318000    1.007939
C       1.775956    3.864480    0.856441
C       2.750199    4.390718    0.082050
N       0.400410    2.050947    1.874520
C      -0.673620    2.887773    2.217675
C      -0.481314    4.274104    1.679465
C       0.662822    4.738324    1.171850
C      -2.134183    2.299785    1.826814
C       2.724670   -2.771725   -0.926844
O       3.494368   -3.700509   -1.229740
O       2.378818   -2.635160    0.364478
C      -1.271173   -2.443819    2.301180
O      -1.545879   -3.633951    2.505189
O      -0.253338   -1.885463    3.017654
C      -2.827297    0.991613    3.925015
O      -1.862588    0.456006    4.608446
O      -3.865125    1.575504    4.557635
C      -0.440271    2.896743    3.745151
O      -0.172573    1.808850    4.399629
O      -0.516699    4.073716    4.417553
C       2.870562    1.799598    1.810847
O       3.505223    2.519022    2.609866
O       3.202055    0.480621    1.734079
H       3.761737   -2.023980   -5.056978
H       2.128828   -3.805684   -4.782703
H      -1.358162   -4.854434   -1.604992
```

H	-0.266704	4.754998	3.759238
H	-2.856283	3.094815	2.064480
H	-2.135975	2.204272	0.733405
H	-4.955860	1.242051	2.470886
H	-5.051801	-3.109239	0.739469
H	-3.381610	-4.615986	-0.297874
H	4.622123	0.150933	-1.774390
H	4.752972	0.031923	-4.347907
H	-4.373869	0.871182	0.900090
H	-1.336902	4.941170	1.706054
H	0.701153	5.757969	0.803730
H	2.815126	5.458086	-0.096961
H	4.273133	4.060457	-1.428783
C	0.764109	-4.139111	-3.066499
C	-4.528261	-0.857215	2.205409
C	3.404779	1.735648	-2.562195
H	-3.772497	1.358062	5.503184
H	1.194091	-5.033229	-2.599525
H	-5.560073	-1.050150	1.886642
H	-4.494004	-1.069670	3.280864
H	4.276642	2.253359	-2.983194
H	2.595015	1.864464	-3.293456
H	0.038459	-4.479188	-3.816819
Fe	1.881659	-0.881157	0.977710
Fe	-0.194334	0.090658	3.097015
C	0.473163	2.022848	-1.224819
O	0.165857	3.070723	-1.801537
O	-0.350806	0.953991	-1.184925
H	0.160295	0.194145	-0.678005
C	0.019573	-1.020815	-3.085177
O	0.498690	-0.048039	-3.652909
O	-1.076817	-1.548086	-3.708316
H	-1.308867	-0.738733	-4.213444
C	-2.577486	-0.654082	-1.060224
O	-3.154957	0.395033	-0.803629
O	-2.918081	-1.238552	-2.255304
H	-2.156061	-1.831647	-2.443128
O	2.516226	-1.880431	2.772550
H	2.467309	-2.832403	2.585159

9. References

(1) Fears, K. P. Measuring the pK/pI of biomolecules using X-ray photoelectron spectroscopy. *Anal. Chem.* **2014**, *86* (17), 8526-8529. DOI: 10.1021/ac5020386.

(2) Christensen, A. S.; Kubar, T.; Cui, Q.; Elstner, M. Semiempirical Quantum Mechanical Methods for Noncovalent Interactions for Chemical and Biochemical Applications. *Chem. Rev.* **2016**, *116* (9), 5301-5337. DOI: 10.1021/acs.chemrev.5b00584.

(3) Cundari, T. R.; Deng, J. PM3(tm) Analysis of Transition-Metal Complexes. *J. Chem. Inf. Comput. Sci.* **1999**, *39* (2), 376-381. DOI: 10.1021/ci980145d.

(4) Wang, G.; Huang, R.; Zhang, J.; Mao, J.; Wang, D.; Li, Y. Synergistic Modulation of the Separation of Photo-Generated Carriers via Engineering of Dual Atomic Sites for Promoting Photocatalytic Performance. *Adv. Mater.* **2021**, *33* (52), e2105904. DOI: 10.1002/adma.202105904.

(5) Xia, T.; Gong, W.; Chen, Y.; Duan, M.; Ma, J.; Cui, X.; Dai, Y.; Gao, C.; Xiong, Y. Sunlight-Driven Highly Selective Catalytic Oxidation of 5-Hydroxymethylfurfural Towards Tunable Products. *Angew. Chem.* **2022**, *61* (29), e202204225. DOI: 10.1002/anie.202204225.

(6) Fan, X.; Ma, J.; Wang, M.; Gao, M.; Xu, J. Selective Aerobic Oxidation of Hydroxyl Compounds Catalyzed by Dimeric N-Salicylidene Oxovanadium Complexes. *ACS Catal.* **2024**, *14* (14), 10538-10548. DOI: 10.1021/acscatal.4c02766.

(7) Zhao, L.; Yang, P.; Shi, S.; Zhu, G.; Feng, X.; Zheng, W.; Vlachos, D. G.; Xu, J. Activation of Molecular Oxygen for Alcohol Oxidation over Vanadium Carbon Catalysts Synthesized via the Heterogeneous Ligand Strategy. *ACS Catal.* **2022**, *12* (24), 15249-15258. DOI: 10.1021/acscatal.2c04601.

(8) Zhu, Y.; Deng, W.; Tan, Y.; Shi, J.; Wu, J.; Lu, W.; Jia, J.; Wang, S.; Zou, Y. In Situ Topochemical Transformation of $ZnIn_2S_4$ for Efficient Photocatalytic Oxidation of 5-Hydroxymethylfurfural to 2,5-Diformylfuran. *Adv. Funct. Mater.* **2023**, *33* (45), 2304985. DOI: 10.1002/adfm.202304985.

(9) Wang, Y.; Liu, H.; Lv, T.; Jia, W.; Zhang, R.; Peng, L.; Zhang, J. Synergetic Effect of Heterojunction and Sulfur Vacancy on $ZnIn_2S_4/CeO_2$ to Enhance the Photocatalytic Performance of 5-Hydroxymethylfurfural into 2,5-Diformylfuran. *Adv. Funct. Mater.* **2024**, 2415842. DOI: 10.1002/adfm.202415842.

(10) Chen, L.; Zhang, T.; Cheng, H.; Richards, R. M.; Qi, Z. A microwave assisted ionic liquid route to prepare bivalent Mn_5O_8 nanoplates for 5-hydroxymethylfurfural oxidation. *Nanoscale* **2020**, *12* (34), 17902-17914, 10.1039/D0NR04738D. DOI: 10.1039/D0NR04738D.

(11) Liao, X.; Guo, M.; Tang, W.; Liu, C.; Luo, W.; Tan, L.; Noguchi, T. G.; Yamauchi, M.; Zhao, Y.; Li, X. Bimetallic single atom promoted α-MnO_2 for enhanced catalytic oxidation of 5-hydroxymethylfurfural. *Green Chem.* **2022**, *24* (21), 8424-8433, 10.1039/D2GC01769E. DOI: 10.1039/D2GC01769E.

(12) Fang, R.; Luque, R.; Li, Y. Selective aerobic oxidation of biomass-derived HMF to 2,5-diformylfuran using a MOF-derived magnetic hollow Fe–Co nanocatalyst. *Green Chem.* **2016**, *18* (10), 3152-3157, 10.1039/C5GC03051J. DOI: 10.1039/C5GC03051J.

(13) Boonyakarn, T.; Wiesfeld, J. J.; Asakawa, M.; Chen, L.; Fukuoka, A.; Hensen, E. J. M.; Nakajima, K. Effective Oxidation of 5-Hydroxymethylfurfural to 2,5-Diformylfuran by an Acetal Protection Strategy. *ChemSusChem* **2022**, *15* (7), e202200059. DOI: 10.1002/cssc.202200059.

(14) Buonerba, A.; Impemba, S.; Litta, A. D.; Capacchione, C.; Milione, S.; Grassi, A. Aerobic Oxidation and Oxidative Esterification of 5-Hydroxymethylfurfural by Gold Nanoparticles Supported on Nanoporous Polymer Host Matrix. *ChemSusChem* **2018**, *11* (18), 3139-3149. DOI: 10.1002/cssc.201801560.

(15) Artz, J.; Mallmann, S.; Palkovits, R. Selective Aerobic Oxidation of HMF to 2,5-Diformylfuran on Covalent Triazine Frameworks-Supported Ru Catalysts. *ChemSusChem* **2015**, *8* (4), 672-679. DOI: 10.1002/cssc.201403078.

(16) Zhang, Z.; Yuan, Z.; Tang, D.; Ren, Y.; Lv, K.; Liu, B. Iron Oxide Encapsulated by Ruthenium Hydroxyapatite as Heterogeneous Catalyst for the Synthesis of 2,5-Diformylfuran. *ChemSusChem* **2014**, *7* (12), 3496-3504. DOI: 10.1002/cssc.201402402.

(17) Ma, J.; Du, Z.; Xu, J.; Chu, Q.; Pang, Y. Efficient aerobic oxidation of 5-hydroxymethylfurfural to 2,5-diformylfuran, and synthesis of a fluorescent material. *ChemSusChem* **2011**, *4* (1), 51-54. DOI: 10.1002/cssc.201000273.

(18) Liu, H.; Cao, X.; Wei, J.; Jia, W.; Li, M.; Tang, X.; Zeng, X.; Sun, Y.; Lei, T.; Liu, S.; et al. Efficient Aerobic Oxidation of 5-Hydroxymethylfurfural to 2,5-Diformylfuran over Fe_2O_3-Promoted MnO_2 Catalyst. *ACS Sustainable Chem. Eng.* **2019**, *7* (8), 7812-7822. DOI: 10.1021/acssuschemeng.9b00010.

(19) Mishra, D. K.; Cho, J. K.; Kim, Y. J. Facile production of 2,5-diformylfuran from base-free oxidation of 5-hydroxymethyl furfural over manganese–cobalt spinels supported ruthenium nanoparticles. *J. Ind. Eng. Chem.* **2018**, *60*, 513-519. DOI: 10.1016/j.jiec.2017.11.040.

(20) Nocito, F.; Ventura, M.; Aresta, M.; Dibenedetto, A. Selective Oxidation of 5-(Hydroxymethyl)furfural to DFF Using Water as Solvent and Oxygen as Oxidant with Earth-

Crust-Abundant Mixed Oxides. *ACS Omega* **2018**, *3* (12), 18724-18729. DOI: 10.1021/acsomega.8b02839.

(21) Zhang, C.; Zhan, P.; Shan, H.; Ren, W.; Liu, Y.; Liu, X.; Zheng, S.; Liao, Z.; Cai, D.; Qin, P. Research on highly efficient photocatalytic selective oxidation of HMF to DFF using CdS@MXene Composites: Construction of Schottky junctions and mechanistic investigation. *Chem. Eng. J.* **2025**, *506*. DOI: 10.1016/j.cej.2025.159946.

(22) Halgren, T. A. Merck molecular force field. I. Basis, form, scope, parameterization, and performance of MMFF94. *J. Comput. Chem.* **1996**, *17* (5-6), 490-519. DOI: 10.1002/(sici)1096-987x(199604)17:5/6<490::Aid-jcc1>3.0.Co;2-p.
